# Genome-wide identification and evolution of WNK kinases in Bambusoideae and transcriptional profiling during abiotic stress in *Phyllostachys edulis*

**DOI:** 10.7717/peerj.12718

**Published:** 2022-01-13

**Authors:** RongXiu Liu, Naresh Vasupalli, Dan Hou, Antony Stalin, Hantian Wei, Huicong Zhang, Xinchun Lin

**Affiliations:** 1 State Key Laboratory of Subtropical Silviculture, Zhejiang A & F University, Lin’an, Zhejiang, China; 2 State Key Laboratory of Subtropical Silviculture, Department of Traditional Chinese Medicine, Zhejiang A & F University, Lin’an, Zhejiang, China

**Keywords:** WNK, Moso bamboo, Gene expression, Abiotic stress

## Abstract

With-no-lysine (WNK) kinases play vital roles in abiotic stress response, circadian rhythms, and regulation of flowering time in rice, Arabidopsis, and Glycine max. However, there are no previous reports of WNKs in the Bambusoideae, although genome sequences are available for diploid, tetraploid, and hexaploid bamboo species. In the present study, we identified 41 *WNK* genes in five bamboo species and analysed gene evolution, phylogenetic relationship, physical and chemical properties, *cis*-elements, and conserved motifs. We predicted the structure of PeWNK proteins of moso bamboo and determined the exposed, buried, structural and functional amino acids. Real-time qPCR analysis revealed that *PeWNK5*, *PeWNK7*, *PeWNK8*, and *PeWNK11* genes are involved in circadian rhythms. Analysis of gene expression of different organs at different developmental stages revealed that *PeWNK* genes are tissue-specific. Analysis of various abiotic stress transcriptome data (drought, salt, SA, and ABA) revealed significant gene expression levels in all *PeWNKs* except *PeWNK11*. In particular, *PeWNK8* and *PeWNK9* were significantly down- and up-regulated, respectively, after abiotic stress treatment. A co-expression network of *PeWNK* genes also showed that *PeWNK2*, *PeWNK4*, *PeWNK7*, and *PeWNK8* were co-expressed with transcriptional regulators related to abiotic stress. In conclusion, our study identified the *PeWNKs* of moso bamboo involved in circadian rhythms and abiotic stress response. In addition, this study serves as a guide for future functional genomic studies of the *WNK* genes of the Bambusoideae.

## Introduction

Protein kinase is a large superfamily of enzymes known to phosphorylate the threonine, tyrosine, and serine residues of target proteins (Kumar, Raina & Sultan, 2020). They constitute about 4% of the *Arabidopsis thaliana* proteome and are involved in various functions such as development, cell cycle and signal transduction (Manuka, Karle & Kumar, 2019; Manuka, Saddhe & Kumar, 2015; Wang et al., 2008). A unique subfamily of serine/threonine protein kinases related to the STE20/PAK-like family is called With-no-lysine (WNK) kinases and is found only in multi-cellular organisms (Kumar et al., 2011; Xu et al., 2000). The WNK kinases contain a conserved lysine residue in the subdomain II within the N-terminal domain, which is essential for ATP binding. However, this conserved lysine residue in the active site is absent in the WNK subdomain II (Xu et al., 2000). Moreover, the lysine in subdomain-I is involved in kinase phosphorylation, and it is the characteristic feature of the WNK family (McCormick & Ellison, 2011).

In plants, *WNK* genes are involved in physiological functions such as maintenance of circadian cycle, root architecture, signal transduction, response to abiotic stress, and flowering time by affecting photoperiod (Kahle et al., 2006; Urano et al., 2015; Urano et al., 2012; Wang et al., 2010). Currently, 11 *WNKs* are known in *A. thaliana* and nine *WNKs* in rice, but only a few genes have been well studied (Manuka, Saddhe & Kumar, 2015). For example, AtWNK1 phosphorylates APRR3 protein, the part of APRR1/TOC1 quintet associated with the clock, to regulate circadian rhythms (Nakamichi et al., 2002). At the same time, the involvement of *AtWNK2*, *AtWNK4*, and *AtWNK6* in circadian rhythms has also been reported (Nakamichi et al., 2002). Similarly, *OsWNK1* shows a rhythmic expression profile under circadian and diurnal conditions and responds to abiotic stress in rice (Kumar et al., 2011).

Furthermore, a knock-out study has demonstrated the importance of *AtWNK8* in abiotic stress (Zhang et al., 2013) and overexpression of *AtWNK9* increases drought tolerance through the ABA signaling cascade (Xie et al., 2014). In addition, nine *WNK(1-9)* have been identified in rice that exhibits differential transcriptional regulation for different abiotic stresses such as heat, cold, salt, and drought (Manuka, Saddhe & Kumar, 2015). At the same time, overexpression of *OsWNK9* enhances the tolerance to salt, drought, and arsenite in *A. thaliana* (Manuka et al., 2021; Xu et al., 2000). Similarly, root-specific *GmWNK1* in *Glycine max* regulates root system architecture and stress response *via* an ABA-dependent signaling pathway (Rodan & Jenny, 2017). At the same time, overexpression of *GmWNK1* in *A. thaliana* showed tolerance towards osmatic and salt stress (Wang et al., 2011). In addition, a total of 114 *WNKs* were identified from eight fruit tree species. It was predicted that *PpWNK.A2* and *PpWNK.E3.1* genes might be related to early fruit development, while *PpWNK.A1* is likely associated with fruit ripening (Cao et al., 2019).

Bamboos (Bambusoideae) are among the fastest-growing plants globally, and *Phyllostachys edulis* (moso bamboo) is the most widespread bamboo species in China and has high economic value as edible shoots, timber, and pulp (Choudhury, Sahu & Sharma, 2012). Bamboo can be divided into four monophyletic lineages based on the level of ploidy: diploid herbaceous bamboo, tetraploid temperate and neotropical woody bamboo, and hexaploid paleotropical woody bamboo. Recently, Zhao et al. (2018) reported the chromosome level *P. edulis* (temperate tetraploid woody bamboo) whole-genome sequence. At the same time, Guo et al. (2019) reported the draft genome sequences of *Olyra latifolia* and *Raddia guianensis* (diploid herbaceous bamboo), *Guadua angustifolia* (tetraploid neotropical woody bamboo) and *Bonia amplexicaulis* (hexaploid paleotropical woody bamboo). Due to climate change, naturally growing bamboo species were subjected to different kinds of abiotic stress. Recently, Liu et al. (2019) reported that the *P. edulis* yield and the quality of winter shoots were severely affected by abiotic stress conditions. Therefore, studying the genes involved in abiotic stress in bamboo species is helpful to develop better adapted genetically modified bamboo plants to the changing environment. The availability of the chromosome level genome of *P. edulis*, draft genome sequences of other bamboo species, and various transcriptomic data from tissues provide the opportunity for genome-wide analysis *WNK* genes (Guo et al., 2019; Zhao et al., 2018). In this study, we identified 41 *WNK* genes belonging to the five bamboo species. Then, we analysed the physicochemical properties, protein structure, and evolution of the WNKs of the Bambusoideae. We also analysed the expression of *PeWNKs* genes in different tissues, the response to abiotic stress, and the co-expression network. The present study results provide a basis for the functional analysis of *WNK* genes in *P. edulis*.

## Materials & Methods

### Plant materials

*P. edulis* seeds used for transcriptomic data were collected in Linchuan County, Guangxi Zhuang Autonomous Region, China. For qPCR analysis, *P. edulis* leaves were collected from the Cuizhu Garden of Zhejiang Agriculture and Forestry University. Samples were collected every four hours, from 6 AM on April 25, 2021, to 48 h.

### Identification of *WNK* genes from *P. edulis* genome databases

The *WNK* genes of *A. thaliana* and rice were downloaded from the Phytozome (https://phytozome.jgi.doe.gov/pz/portal.html). We used the genome database of *P. edulis* and transcriptomic data (Zhao et al., 2018) to identify *WNK* family genes through the local BLAST analysis. At the same time, other *WNK* genes of the Bambusoideae were isolated from draft sequences of the herbaceous diploid bamboo species *O. latifolia* and *R. guianensis* and the tetraploid and hexaploid woody species *G. angustifolia* and *B. amplexicaulis* (Guo et al., 2019). The candidate genes obtained were verified against the NCBI database (https://www.ncbi.nlm.nih.gov/). The amino acid sequences of *WNK* genes were aligned to confirm conserved regions. The sequences without a complete reading frame and conserved domain were removed.

### Physicochemical properties, phylogenetic tree and motif analysis of *WNK* genes

The amino acid number, molecular weight, and isoelectric point of PeWNK proteins were calculated using the online software ExPASy (https://www.ExPASy.org/). The phylogenetic tree was constructed using the maximum-likelihood method with MEGA-X (Kumar et al., 2018). The conserved domains of plant species *A. thaliana*, *Glycine max*, *Oryza sativa*, *Zea mays*, *P. edulis*, *R. guianensis*, *O. latifolia*, *G. angustifolia* and *B. amplexicaulis* were used to construct the phylogenetic tree. A bootstrap value of 1,000 replicates was calculated to evaluate the statistical significance of clade level relationships. Subsequently, the phylogenetic tree for WNKs was imported into the ITOL server (http://itol.embl.de/). The conserved motifs were identified using the MEME server and visualized in TBtools (Chen et al., 2020).

### Protein secondary and tertiary structure of *PeWNK* genes

The secondary structures of the WNK proteins of *P. edulis* were predicted through the online website SOPMA (https://npsa-prabi.ibcp.fr/NPSA/npsa_sopma.html) with the default parameters of four conformational states (helix, sheet, turn, coil) and similarity threshold eight. The tertiary structures of the WNK proteins of *P. edulis* were predicted using the Modeller tool with the help of the Consurf server (Berezin et al., 2004). The models of the proteins were built based on the ’ConSeq’ mode and the given selected parameters were used to build the multiple sequence alignments. The homologs were taken from the UniProt database and CS-BLAST was used as the algorithm for homolog search (CSI-BLAST E-value: 0.0001; No. of CSI-BLAST Iterations: 3; maximal percentage ID between sequences: 95; minimal percentage ID for homologs: 35; 150 sequences querying the list of homologs for retrieval. For phylogenetic tree analysis, Neighbor-Joining with ML distance algorithm was used. Bayesian computational calculation and best-fit model of substitution for proteins were used to calculate the conservation scores.

### Analysis of *Cis*-acting element

We retrieved the upstream sequence region (2 Kb) of the *WNK* genes from the genome database to analyse the *cis*-acting elements. The retrieved sequences were analysed using the PlantCARE program (http://bioinformatics.psb.ugent.be/webtools/plantcare/html/) to identify the putative *cis*-acting elements. The *cis*-elements related to ABA, GA, SA and circadian rhythms were visualized through TBtools (Chen et al., 2020).

### The *PeWNK* gene expression in different tissues

Transcriptome data of 26 different tissues of *P. edulis* were obtained from the NCBI Short Read Archive database (SRX2408703) (Zhao et al., 2018) and used for tissue expression studies. The FPKM values of the *WNK* genes of *P. edulis* were used to develop a heat map using TBtools (Chen et al., 2020).

### Expression analysis of *PeWNK* genes in response to abiotic stress

Thirty-day old equal height *P. edulis* seedlings were used for abiotic stress treatment. Seedlings were treated with 25% polyethylene glycol (PEG), 200 µM Abscisic acid (ABA), 1 mM salicylic acid (SA) (unpublished) and 200 mM sodium chloride (NaCl) (Yang et al., 2010) nutrient solution for 3 h and 24 h, respectively. Total RNA was isolated from young leaves and RNAseq data were generated on the Illumina platform (pair-end reads) in three biological and technical replicates (GSE169067). The adapter sequences and low-quality reads were removed and the high-quality reads were mapped to the reference genome sequence using the Hisat2 tool. FPKM values of the RNAseq data were developed and used to generate graphs.

### Real-time qPCR analysis

Total RNA from leaf samples was isolated using Trizol reagent. According to the manufacturer’s instructions, cDNA was synthesised using the PrimeScript RT reagent kit with gDNA Eraser (TaKaRa, Shiga, Japan). The 2XNovoStart SYBR qPCR SuperMix Plus (novoprotein, Suzhou, China) was used for qRT-PCR amplification in a real-time PCR instrument (BioRad, USA). The qPCR reaction conditions are as follows: initial denaturation 95 °C for 5 min, followed by 40 cycles of 30 s at 94 °C, 30 s at 60 °C, and 30 s at 72 °C. A melting curve was included from 65 to 95 °C to check amplification specificity. The 2−ΔΔCt method was used to determine the relative expression levels. In addition, *NTB* was used as a reference gene in *P. edulis* according to previous studies (Zhao et al., 2019). The qPCR primers for the *PeWNK* genes used for gene expression analysis are listed in Table S1.

### Co-expression analysis of *PeWNK* genes

We submitted the *PeWNK* genes to the BambooNET (http://bioinformatics.cau.edu.cn/bamboo/index.html) and acquired the co-expression network data.

## Results

### Identification of the Bambusoideae *WNK* genes

The genome database of *P. edulis* and the draft genomes of *R. guianensis*, *O. latifolia*, *G. angustifolia*, and *B. amplexicaulis* were used to find the *WNK* candidate genes in the Bambusoideae. In addition, the *WNK* genes of *A. thaliana* and rice were downloaded from Phytozome and used as reference genes to identify the *WNK* genes of the Bambusoideae through the local BLASTP. The sequences containing the serine/threonine-protein kinase domain are referred to as Bambusoideae *WNK* genes (*PeWNKs*, *RguWNKs*, *OlaWNKs*, *GanWNKs*, and *BamWNKs*) (File S1). A total of 11 *WNK* genes of *P. edulis* (*PeWNK1-11*) and 30 *WNK* genes of the other four bamboo species were identified. The WNK proteins of the Bambusoideae range from 257 to 1905 amino acids, of which RguWNK1 is the smallest and PeWNK8 is the largest. At the same time, the molecular weight is 29047.42 and 157,857.24, respectively. Moreover, the isoelectric point and instability index are 4.56 to 6.74 and 29 to 59.63, respectively. In addition, the aliphatic index and the grand average of hydropathicity are 19.42 to 95.95 and −0.647 to 0.977, respectively (Table S2). Furthermore, we identified that *PeWNK* genes were located on nine scaffolds, with scaffolds 4 and 10 containing two genes, whereas the remaining scaffolds contained only one gene (Fig. S1).

**Figure 1:**
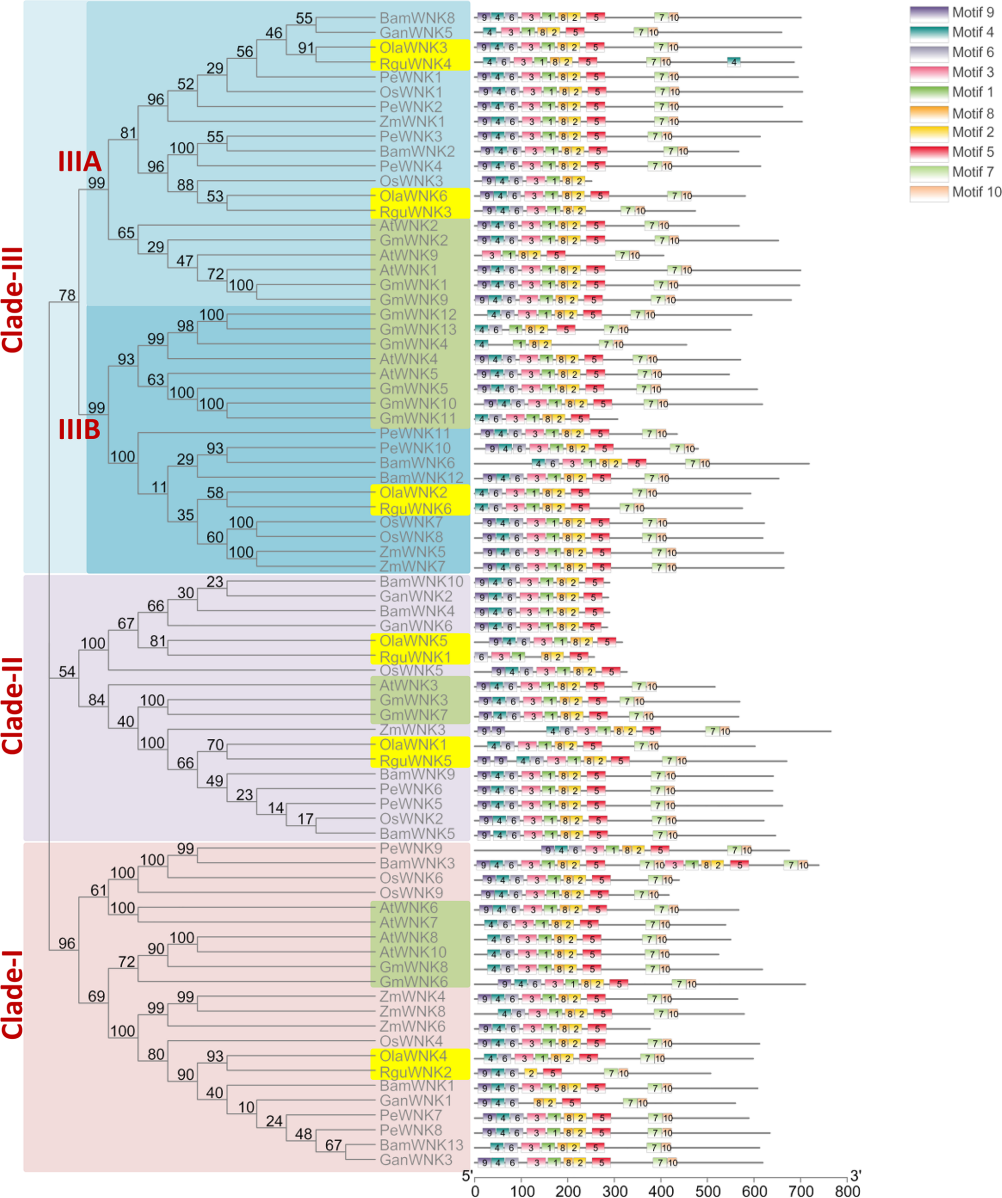
The phylogenetic tree of *WNK* genes from dicot and monocot plants. The phylogenetic tree was constructed using *WNK* sequences of *Arabidopsis thaliana* (At), Glycine max (Gm), *Oryza sativa* (Os), *Zea mays* (Zm), *P. edulis* (Pe), *O. latifolia* (Ola), *R. guianensis* (Rgu), *G. angustifolia* (Gan) and *B. amplexicaulis* (Bam). The bootstrap support values were mentioned as the numbers on the branches. Clade I, II and III are indicated in the blue, violet and pink colours, respectively. The dicot plants *WNK* genes were indicated in the grey colour boxes and the diploid bamboo species are indicated in the yellow colour boxes. The conserved motifs (1–10) are mentioned in different colour boxes.

### Evolution of *WNK* gene family

To understand the evolution of *WNK* genes, a total of 78 *WNK* genes (*AtWNKs*, *GmWNKs*, *OsWNKs*, *ZmWNKs*, *PeWNKs*, *BamWNKs*, *GanWNKs*, *OlaWNK,* and *RguWNKs*) were used to construct the phylogenetic tree (File S1). The highly aligned peptide sequences were used to generate a phylogenetic tree using the maximum likelihood method with 1,000 bootstrap replicates (Fig. 1). The *WNKs* were mainly divided into three clades, namely clade I, II and III. In addition, clade III was divided into clades IIIA and IIIB and clade III has more genes than clades I and II. Additionally, all clades were supported by high bootstrap values. Based on the topological structure, the evolution of *WNK* genes was clearly divided between monocots and dicots in the phylogenetic tree. In clades, I, II, IIIA, and IIIB, monocot and dicot *WNK* genes were divided into two sub-branches with higher bootstrap values. These results suggest that *WNKs* were present before the divergence of monocot and dicot plants. Moreover, the *OlaWNKs* and *RguWNKs* of herbaceous bamboo were also divided into sub-branches compared with the other woody bamboo species. This suggests that *WNKs* evolved separately after polyploidisation in the *Bambusoideae* (Fig. 1).

The evolution of plant species is driven by polyploidisation, including in the Bambusoideae (Ramakrishnan et al., 2020). In the phylogenetic tree, the *WNKs* of diploid and polypoid bamboo species were also separated by sub-braches. Moreover, the copy number of *WNKs* was increased in the tetraploid *P. edulis* and hexaploid *B. amplexicaulis* compared to the diploid bamboo species *O. latifolia* and *R. guianensis*. In contrast, the copy number of *WNKs* is surprisingly lower in the tetraploid *G. angustifolia* than in the diploid bamboo species. Furthermore, we analysed the evolution of specific domains in WNKs between dicot and monocot plants (Fig. 1). Using the MEME server, we identified ten conserved motifs in the WNKs proteins. With few exceptions, most WNKs in all three clades contain all ten domains in the same serial order. *RguWNKI* in clade II and *GmWNK4* in clade III contain the least number of six domains. *BamWNK3* in clade I, on the other hand, has 17 domains, with domains 1, 2, 3, 5, 7, 8, and 10 were duplicated. In addition, the starting domain nine is absent in most of the monocot groups of clade I. In contrast, in clade II, the last two domains 7 and 10 are missing in half of the bamboo *WNKs*.

### *Cis*-acting elements responsive to abiotic stress and circadian rhythm

**Figure 2:**
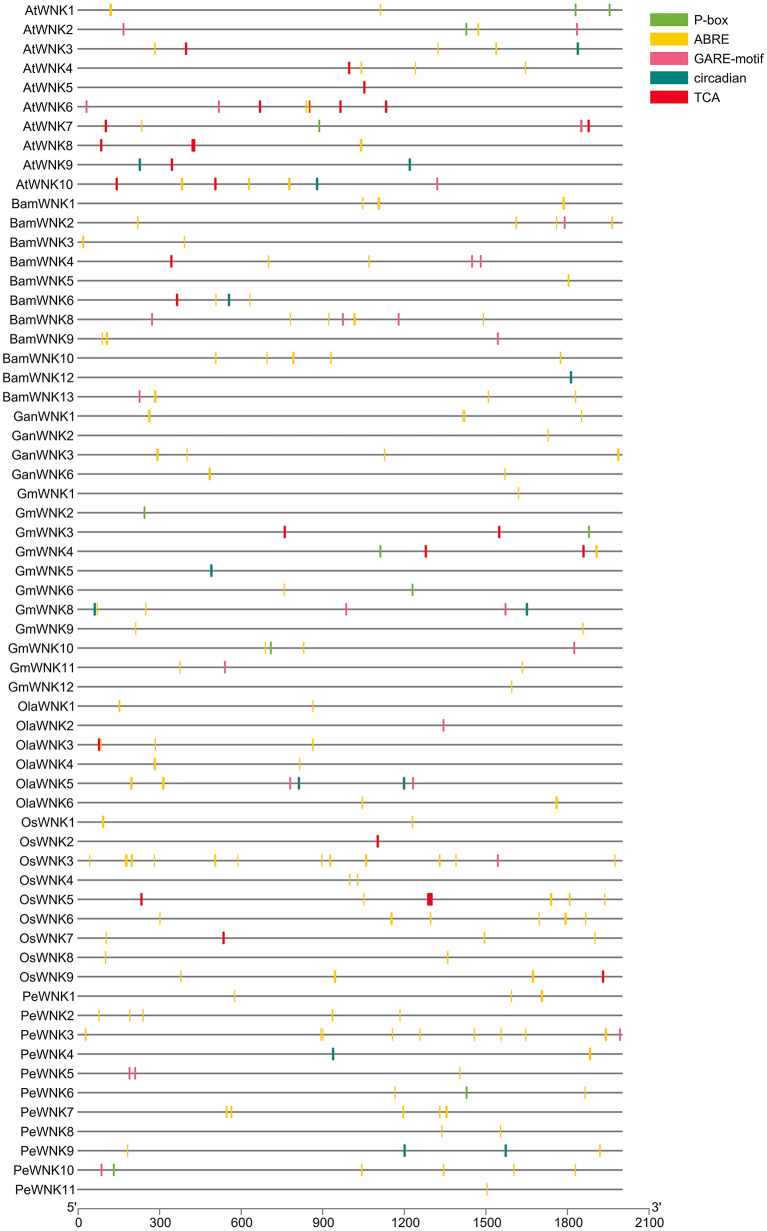
The conserved *cis*-elements analysis of *WNK* genes in the promoter regions of Bambusoideae and other monocot and dicot plants, related to stress response (P-box, ABRE, GARE-motif, TCA and circadian).

*Cis*-acting elements affect genes involved in the stress response. Hence, studying the *cis*-acting elements in the promoter region helps to understand the role of *WNK* genes in the stress response. Therefore, we analysed the putative *cis*-elements in the 2 kb region upstream of the translational start site of *WNK* genes in both monocot (*OsWNKs*, *PeWNKs*, *BamWNKs*, *GanWNKs*, *OlaWNK,* and *RguWNKs*) and dicot (*AtWNKs* and *GmWNKs*) plants (File S2). Among them, we focused on exploring the ABA, GA, SA and circadian rhythm responsive elements, and there are several *cis*-elements associated with them in *WNKs*. For example, ABA-responsive elements (ABREs) are present in 61 genes, including all 11 *PeWNK* genes (File S3). We, therefore, hypothesise that ABA stress responses regulate most *WNK* genes. Moreover, the GA responsive GARE-motif, P-box, and TATA-box elements are present in the promoter regions of 21, 18, and 71 *WNK* genes, respectively. Similarly, SA responsive element TCA is present in the promoter region of 24 *WNK* genes. Interestingly, 12 *WNK* genes also have *cis*-acting elements associated with circadian control (Fig. 2). Further, GC-motif and SP1 are present in some of the monocot *WNK* and *GmWNK* genes but absent in Arabidopsis (Fig. S2).

### Prediction of the protein structure of *P. edulis* WNK

**Figure 3:**
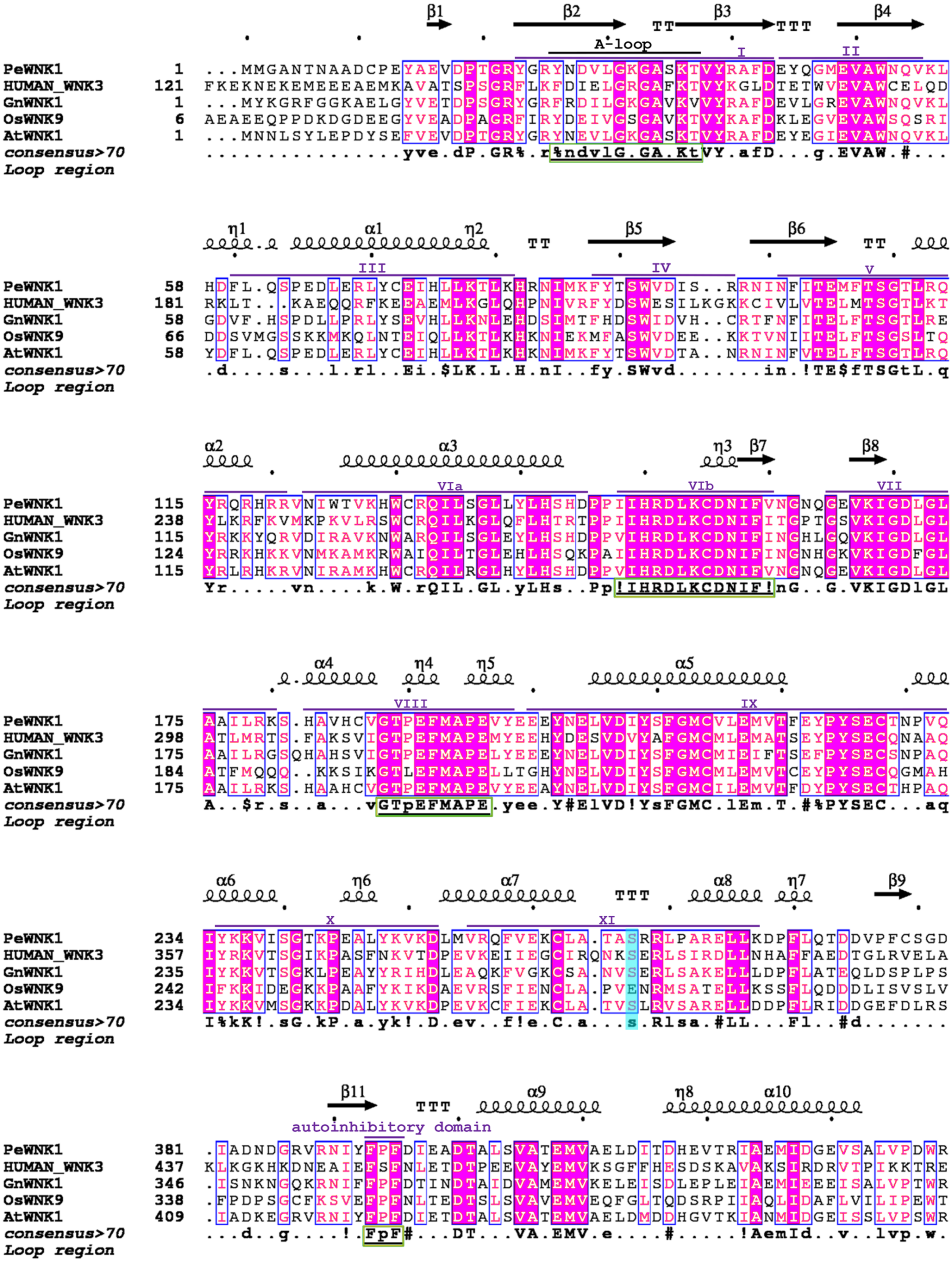
Multiple sequence alignment between PeWNK1, HUMAN_WNK1, GnWNK1, OsWNK9, and AtWNK1 protein sequences. Conserved domains, motif and secondary structural arrangements were highlighted. The phosphorylation sites were mentioned in the blue background.

**Figure 4:**
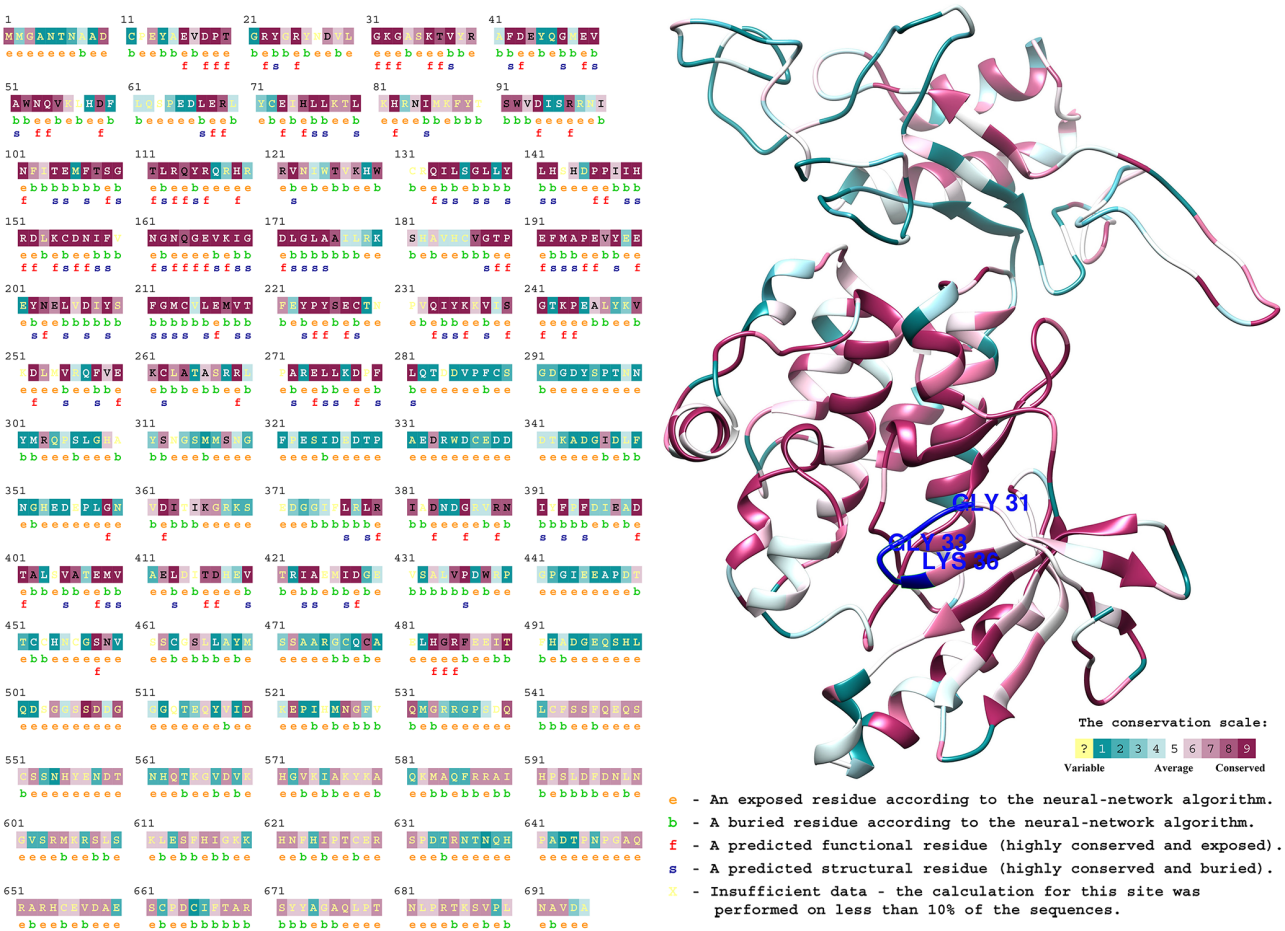
Conserved domain sequence analysis of WNK in the Bambusoideae (PeWNK1) protein predicted by Consurf server. Predicted homology model of PeWNK1 using modeler; highly conserved WNK kinase domain and autoinhibitory domain were highlighted.

The secondary structure of the protein plays an essential role in constructing the tertiary structure of the protein and its normal function. It mainly consists of hydrogen bonds and the primary forms include α-helix, β-turn, random curling, *etc*. The secondary structure of 11 *P. edulis* WNK proteins was predicted using the online website SOPMA (Table 1). It can be seen that the WNK protein of *P. edulis* has a relatively similar protein secondary structure. Modeller 9.19 software was used to predict the tertiary structure of 11 identified WNK proteins of *P. edulis* (Ashkenazy et al., 2016). Furthermore, we compared and analyzed the domains and motifs of PeWNK1 with human_WNK3, GmWNK1, OsWNK9 and AtWNK1 (Fig. 3). The *PeWNK1* sequence was similar to all other previously published *WNKs* genes (Manuka, Saddhe & Kumar, 2018). *PeWNK1* has an N-terminal protein kinase domain divided into 12 subdomains. In addition, an activation loop (A-loop), an autoinhibitory conserved domain-containing FXF motif, the ’IIHRDLKCDNIFI’ motif in subdomain VIb and the ‘GTPEFMAPE’ motif in subdomain VIII were conserved (Fig. 3). Besides, we compared the eleven *WNK* genes from *P. edulis* in which all these A-loops and motifs were conserved (Fig. S3), and we also detected that these A-loops and motifs were conserved in all monocot and dicot plants used in this study (File S1). Moreover, we also analysed the phosphorylation sites of the PeWNK proteins and identified that, except for PeWNK9, all other WNKs contained the phosphorylation sites (Fig. 4 and Fig. S3).

**Table 1:** Protein secondary structure of WNK protein in *P. edulis*.

Protein	ID of gene	Alpha helix	Beta turn	Extended strand	Random coil
PeWNK1	*PH02Gene37861.t1*	36.12%	3.74%	8.92%	51.22%
PeWNK2	*PH02Gene17877.t1*	37.07%	5.75%	11.65%	45.54%
PeWNK3	*PH02Gene03314.t1*	41.44%	3.75%	9.30%	45.51%
PeWNK4	*PH02Gene01510.t1*	38.27%	3.91%	10.10%	47.72%
PeWNK5	*PH02Gene07448.t1*	37.37%	5.30%	11.65%	45.69%
PeWNK6	*PH02Gene25768.t1*	38.12%	4.06%	8.59%	49.22%
PeWNK7	*PH02Gene38251.t1*	37.69%	3.90%	9.85%	48.56%
PeWNK8	*PH02Gene03413.t1*	35.49%	4.57%	11.04%	48.90%
PeWNK9	*PH02Gene20314.t1*	42.60%	5.47%	10.95%	40.98%
PeWNK10	*PH02Gene23702.t1*	40.21%	3.96%	10.83%	45.00%
PeWNK11	*PH02Gene11468.t1*	36.32%	6.21%	16.32%	41.15%

Furthermore, all PeWNK protein sequences were compared with known WNK proteins in the Uniprot database using ConSurf domain analysis (Ashkenazy et al., 2010; Celniker et al., 2013). Based on the phylogenetic relationship between the homologous sequences of WNK, the conserved regions of amino acids were identified. For instance, the conserved domain region of PeWNK1 is shown in the colour magenta (Fig. 4). The remaining conserved domains of the ten PeWNK proteins are listed in a File S4. As mentioned in Fig. 3, most of the amino acids in the activation loop, autoinhibitory domain (FPF), and kinase domain are located in the conserved region. We also identified the exposed, buried, functional and structural (e, b, f, s) residues/amino acids in the PeWNKs. All functional residues are the exposed residues, while all structural residues are buried (Fig. 4).

### *PeWNK* genes response to circadian rhythms

**Figure 5:**
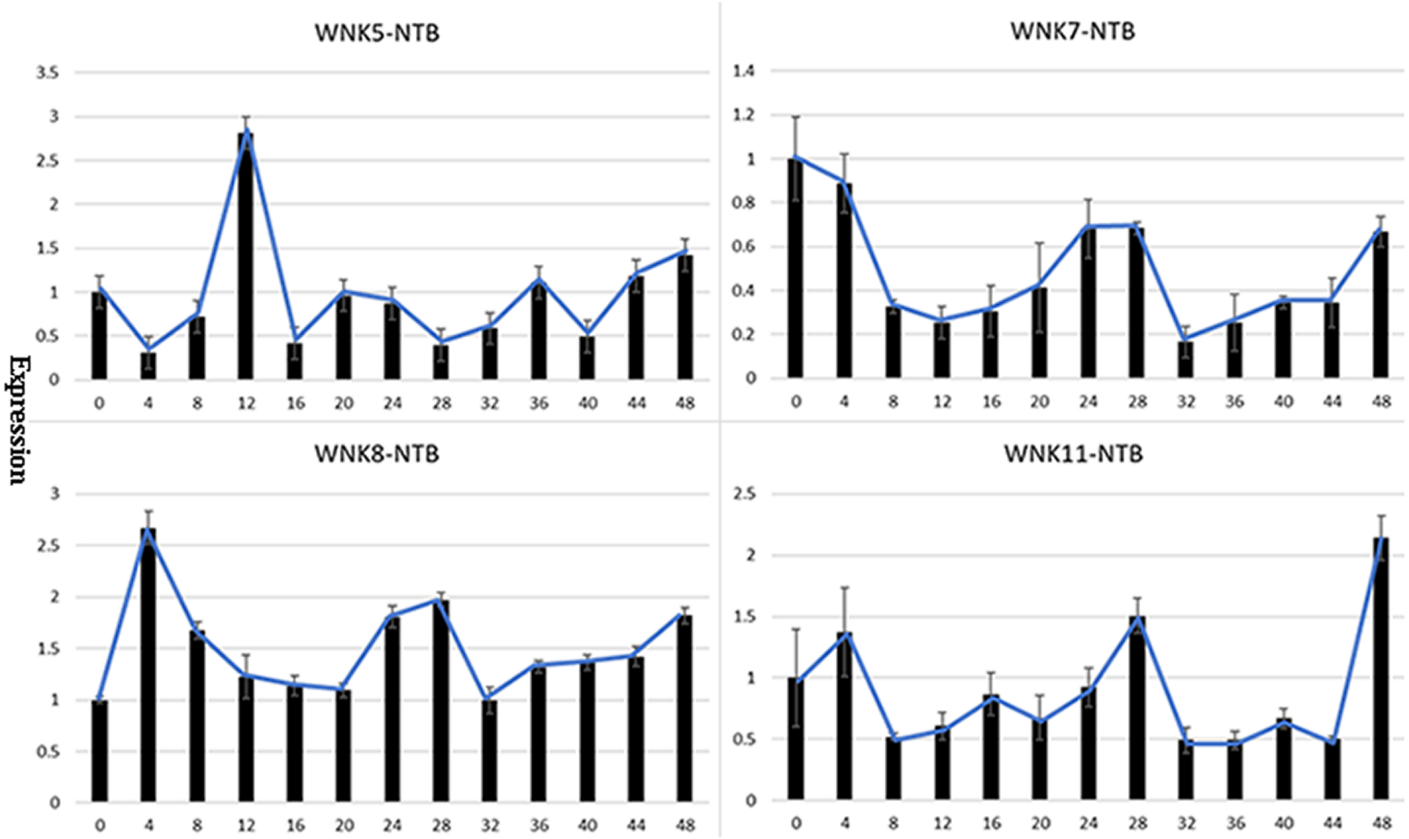
Expression analysis of *PeWNK* genes for the circadian cycle. qRT-PCR analysis of *PeWNK5*, *PeWNK7*, *PeWNK8* and *PeWNK11* genes normalized with *NTB*. Moso bamboo cDNA leaf samples 0–48 h. The error bar indicates the standard deviation (*n* = 3).

*WNK* genes have been previously reported to be involved in circadian rhythms (Kumar et al., 2011; Nakamichi et al., 2002). Therefore, we collected leaf samples of *P. edulis* every four hours starting from 6 AM up to 48 h and conducted qPCR experiments to identify the *PeWNK* genes of *P. edulis* involved in circadian rhythms. The results showed that among the 11 *PeWNK* genes of *P. edulis*, *PeWNK5*, *PeWNK7*, *PeWNK8,* and *PeWNK11* follow circadian rhythms (Fig. 5). The *PeWNK7*, *PeWNK8,* and *PeWNK11* genes show a clear circadian expression pattern in the morning, with a peak forming every 0 and 4 h (6 and 10 AM). In contrast, the expression pattern of *PeWNK5* follows a 12 h cycle. After 0 h in the morning, the expression drops to a very low level at 4 h and increases again at 8 and 12 h (2 and 6 PM) (Fig. 5).

### Expression profile of *PeWNK* genes in different tissues

**Figure 6:**
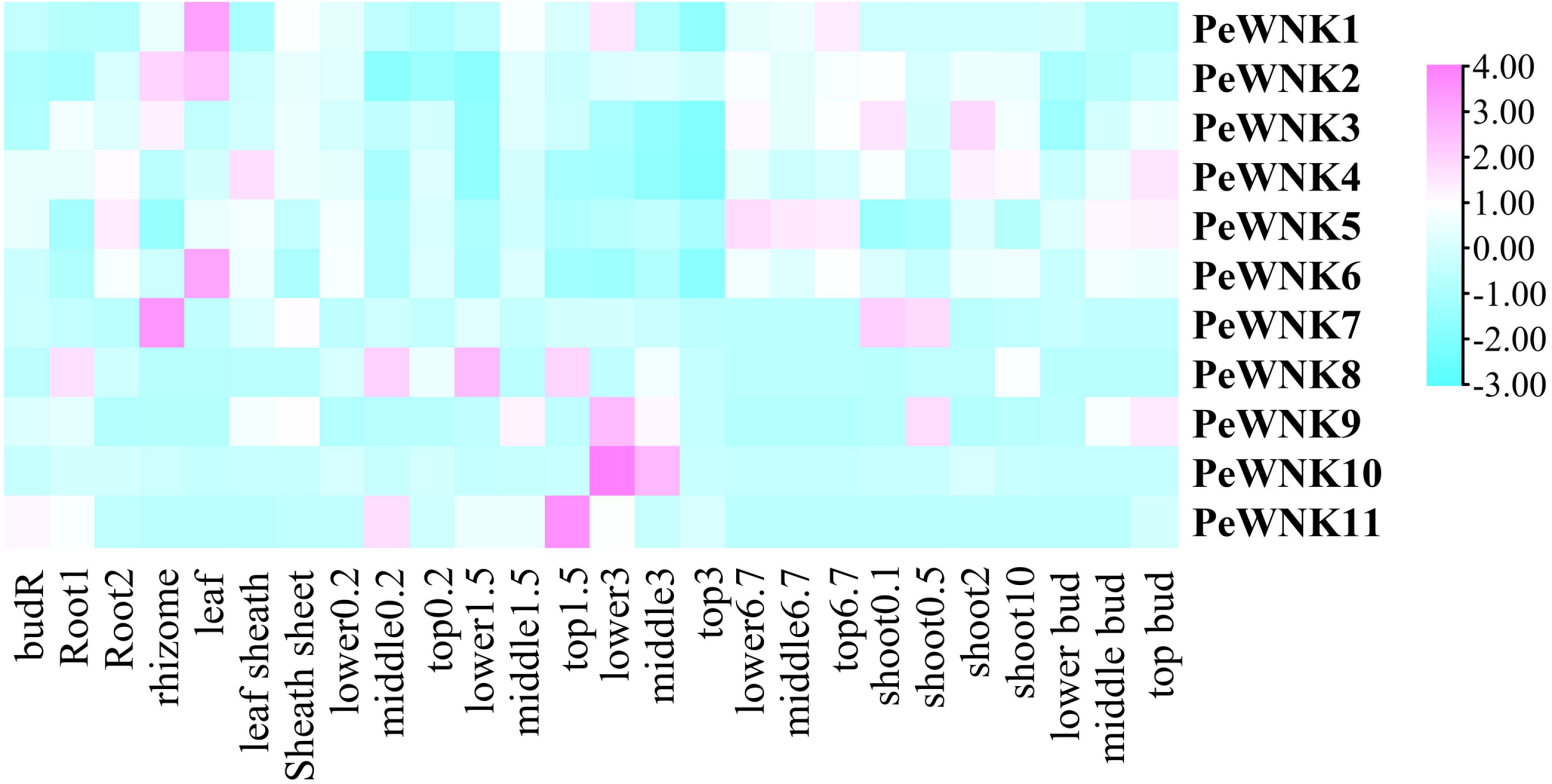
Expression of *PeWNK* genes in 26 different tissues and stages of bamboo growth. The log2 expression values represent each colour box and the colour scale is present on the upper right side.

To elucidate the expression profiles of *PeWNKs* in different tissues, we developed a heatmap using transcriptomic data from 26 different tissues at different developmental stages, as mentioned by Zhao et al. (2018). The heatmap indicates that some *PeWNK* genes have high expression in specific tissues. For example, the expression patterns of *PeWNK10* were very high in the middle and lower portion of the 3 m shoot, while the expression in the other tissues was comparatively low. In addition, *PeWNK7* was expressed in the rhizome, whereas *PeWNK6* and *PeWNK1* were mainly expressed in the leaf. Interestingly, the expression of *PeWNK* genes was relatively low in the rhizome bud (budR), lower bud, and top 3m shoot (Fig. 6).

### Response of *PeWNK* genes under abiotic stress treatments

**Figure 7:**
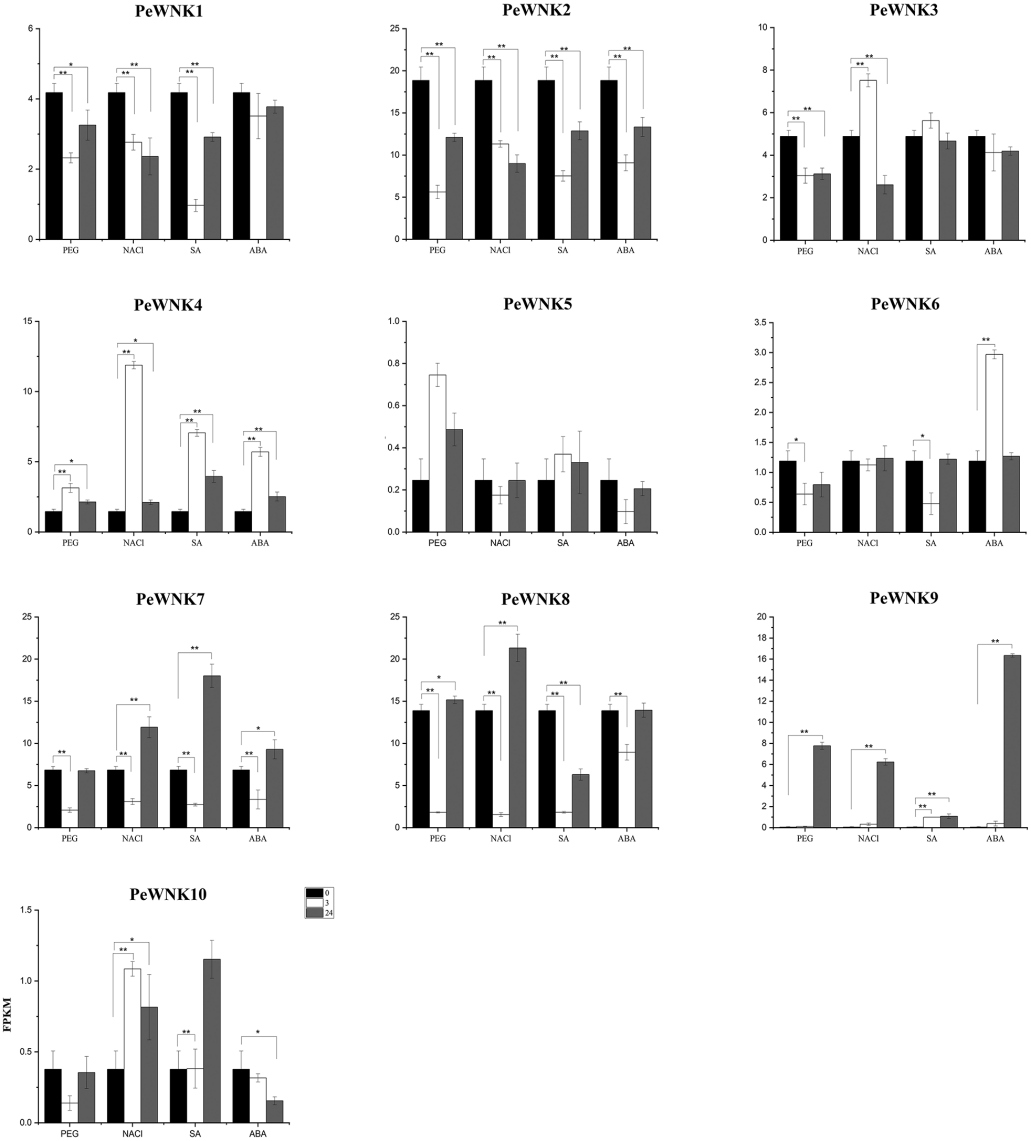
Expression analysis of *PeWNK* genes in response to Polyethylene glycol (PEG), Sodium chloride (NaCl), Abscisic acid (ABA) and Salicylic acid (SA). The FPKM values of transcriptomic data (Moso bamboo seedlings treated with PEG (25%), NaCl (200 mM), ABA (1uM), SA (1 mM) for 3 h and 24 h) are used to develop graphs. The error bar indicates the standard deviation (*n* = 3).

We analysed the transcriptomic data to investigate further the characteristics of *PeWNK* gene expression in *P. edulis* seedlings under drought, salt, SA and ABA treatments. The analyses showed that *PeWNK* genes responded differently at 3 h and 24 h after exposure to drought, salt and hormone stress. In this study, the genes with two-fold differences were considered to be differentially expressed compared with the control (Wang et al., 2020). Among all *PeWNK* genes, the expression of *PeWNK9*, in particular, was significantly up-regulated after abiotic stress treatments (Fig. 7). Under PEG, NaCl and ABA treatment, the relative expression of *PeWNK9* was up-regulated 146, 117, and 307 times respectively, after 24 h compared with the control. Similarly, the relative expression of *PeWNK4* was up-regulated by 2.2–8.2 times of control after 3 h in all treatments. Further, the relative expression of *PeWNK7* and *PeWNK8* was significantly downregulated after 3 h in all treatments. After 24 h of treatment with SA, the expression of *PeWNK7* was up-regulated to 2.6 times and the expression of *PeWNK8* was downregulated to 0.45 times of control (Fig. 7).

After 3 h of treatment with SA, the relative expression of *PeWNK1* was downregulated to 0.23 times of control. Similarly, the expression of *PeWNK2* was significantly downregulated after 3 h of PEG, SA, ABA and 24 h of NaCl treatment. Likewise, the expression of *PeWNK5* and *PeWNK6* was downregulated to 0.4 times after 3 h of SA treatment. While the expression of *PeWNK6* was up-regulated to 2.5 times of control after 3 h of ABA treatment. The expression of *PeWNK10* was significantly up-regulated after both NaCl treatment and 24 h SA treatment. At the same time, expression was downregulated to 0.4 times of control after 3 h treatment with PEG and 24 h treatment with ABA. The expression levels of *PeWNK11* are too low for analysis (Fig. 7).

### Co-expression analysis of *PeWNK* genes

**Figure 8:**
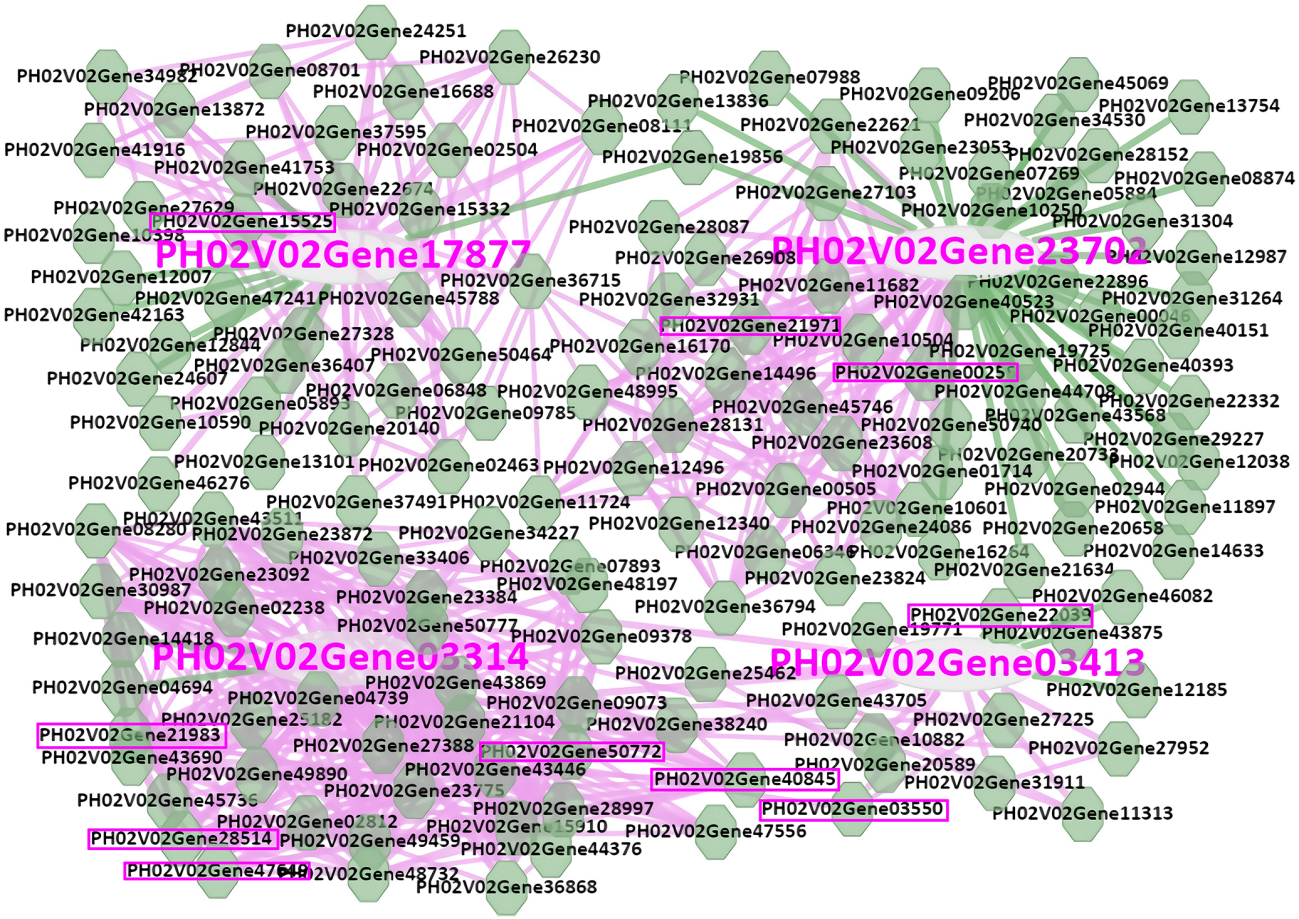
Co-expression network of *PeWNK8* (PH02Gene03413.t1), *PeWNK2* (PH02Gene17877), *PeWNK4* (PH02Gene23702), *PeWNK7* (PH02Gene03314). The boxes indicate the genes involved in abiotic stress response.

A co-expression network has been successfully applied to identify the transcription factors or regulators in many plant species (Bishop et al., 2020; Gao et al., 2020; Yang et al., 2017). To determine the regulators of *PeWNK* genes, we used the BambooNET database. The 11 *PeWNK* genes were searched for transcriptional regulators in the BambooNET database. *PeWNK8* (PH02Gene03413.t1) is co-expressed with 17 genes, including GRAS family transcription factor and F-box protein 2 (Fig. 8). Interestingly, both genes have been reported to be associated with abiotic stress. Similarly, both *PeWNK2* (PH02Gene17877) and *PeWNK4* (PH02Gene23702) were co-expressed with an F-box family protein (PH02Gene00258). Furthermore, *PeWNK7* (PH02Gene03314) is co-expressed with the PEBP (phosphatidylethanolamine-binding protein) family protein and the myb domain protein 48 (File S5). These two proteins are involved in the suppression of flowering and circadian rhythms, respectively.

## Discussion

Bamboo is one of the fastest-growing perennial plants and has the longest vegetative stage before flowering (Liu et al., 2019; Ramakrishnan et al., 2020). However, the mechanisms involved in abiotic stress during bamboo growth are poorly understood. *WNK* genes, which belong to the serine/threonine protein kinases of the STE20/PAK-like subfamily (Manuka, Saddhe & Kumar, 2015) play an essential role in regulating plant salt tolerance and osmotic stress by coordinating ion channels and signal transduction during the transportation process (Kahle et al., 2006; Wang et al., 2010). In addition, *WNK* genes are also involved in circadian rhythms (Nakamichi et al., 2002). To date, *WNK* genes have been identified in Arabidopsis, rice, soya bean, and fruit trees (Cao et al., 2019; Kumar et al., 2011; Wang et al., 2008). However, the identity and function of *WNKs* in bamboo, including *P. edulis*, have not yet been identified. In this study, we identified *WNK* genes in diploid and polyploid bamboo species and investigated the evolution of *WNKs* between monocot and dicot plants. Further, we identified the protein structure, response to abiotic stress, tissue-specific expression, and co-expression analysis of *PeWNK* genes in *P. edulis*.

We identified a total of 41 *WNK* genes from the available bamboo genome database and investigated their gene evolution, physical and chemical properties, and conserved motifs. The putative amino acid lengths of WNKs from Rice, *G. max*, and *Populus trichocarpa* range from 328–705, 480–738 and 297–739 amino acids, respectively (Manuka, Saddhe & Kumar, 2015; Wang et al., 2010). At the same time, human WNK1 has a length of 2,382 amino acids (VerõÂssimo & Jordan, 2001). In our study, the length of the amino acids of WNK of diploid bamboo is 257–702, that of tetraploid bamboo is 285–1905, and that of hexaploid bamboo is 290–739. These results suggest that the amino acid lengths of diploid, hexaploid and tetraploid GanWNKs are similar to those of rice and *G. max*. Interestingly, the amino acid length of the four *PeWNK* genes in *P. edulis* ranges from 1771–1905, which is almost the size of human WNKs and three times longer than OsWNK.

PeWNKs have the N-terminal protein kinase domain, which has the altered lycine residue in the Gly-X-Gly-X-X-Lys-X-Val motif of subdomain I instead of Gly-X-Gly-X-X-Gly-X-Val. In addition, the *WNK* genes of higher plants were divided into three clades. These results are consistent with previous findings in plants and animals (Manuka, Saddhe & Kumar, 2015; Xu et al., 2000). Moreover, the distribution of conserved motifs was similar among WNK proteins in the same clade. These results and phylogenetic analysis support the reliability of clade classification and the similar functions of proteins in the same clade. Moreover, the number of genes in the gene families increased with the duplication events and polyploidization (De Grassi, Lanave & Saccone, 2008; Li et al., 2020). The copy number of *WNKs* was increased in the tetraploid *P. edulis* and hexaploid *B. amplexicaulis* compared to the diploid bamboo species *O. latifolia* and *R. guianensis*. In contrast, the copy number of *WNKs* is lower in the tetraploid *G. angustifolia* than in the diploid bamboo species. These results might be due to low coverage, poor sequencing, and incomplete genome database.

Tissue-specific expression analysis of *OsWNK* genes in rice revealed that most *OsWNK* genes are more highly expressed in roots than in other tissues, indicating the role of *OsWNKs* in root formation and architecture (Manuka, Saddhe & Kumar, 2015). In Arabidopsis, *AtWNK8* is mainly expressed in the hypocotyl, primary root, and pistil (Zhang et al., 2013). At the same time, all other *AtWNK* genes (except *AtWNK6*) are expressed in different tissues and organs at different developmental stages (Wang et al., 2008). In the fruit tree *Prunus persica,* gene expression analysis revealed that *PpWNK.A1* is probably involved in fruit ripening, while *PpWNK.A2* and *PpWNK.E3.1* are associated with early fruit development (Cao et al., 2019). In contrast to rice *OsWNKs*, tissue-specific expression analysis of *PeWNK* genes in our study shows that most *PeWNK* genes are expressed only in a particular tissue at a specific plant height, indicating diverse roles in different developmental stages of the tissues.

Various abiotic stress conditions severely affect *P. edulis* yield and the quality of winter shoots (Liu et al., 2019). Protein kinases in plants play a crucial role in stress-induced signal transduction pathways (Kumar et al., 2013). Our results showed that all *PeWNK* genes responded to abiotic stress, except *PeWNK11*. A T-DNA knock-out mutant study showed that *AtWNK8* was induced after salt and sorbitol stress, and disruption of *AtWNK8* enhances tolerance to NaCl and osmotic stress (Zhang et al., 2013). Moreover, overexpression of *OsWNK9* increases tolerance to salt, drought, and arsenite in transgenic Arabidopsis plants (Manuka, Karle & Kumar, 2019; Manuka et al., 2021). Phylogenetic analysis of the gene family shows that *AtWNK8* and *OsWNK9* are closely related to *PeWNK7*, *PeWNK8,* and *PeWNK9*. Our study also provided evidence that the expression of *PeWNK9* was significantly increased after all abiotic stress treatments. In contrast, the expression of *PeWNK8* significantly decreased considerably after 3 h of PEG, NaCl and SA treatments. Similarly, the *OsWNK1* gene was up-regulated after drought and cold stress and downregulated after salt stress (Kumar et al., 2011). Both *PeWNK1* and *PeWNK2* were similar to *OsWNK1* and both were significantly downregulated after all abiotic stresses studied. These results suggest that these proteins have similar functions and are predominantly involved in abiotic stress response.

In addition, our co-expression network analysis also revealed the relationship between abiotic stress genes and *PeWNK* genes. In this study, *PeWNK8* was found to be co-expressed with transcription factor *GRAS* and F-box protein 2. The transcription factor *OsGRAS23* from rice is involved in drought stress response, and the transcription factor *GRAS* from *Vitis amurensis* induces abiotic stress tolerance in Arabidopsis (Xu & Zhang, 2015; Yuan et al., 2016). Similarly, an F-box protein MAX2 regulates drought tolerance in Arabidopsis (Bu et al., 2014). Interestingly, *PeWNK8* was downregulated after PEG, NaCl and SA treatments, indicating its involvement in the abiotic stress response.

## Conclusions

In the present study, we identified 41 *WNK* genes in five Bambusoideae species and analyzed the conserved motifs, domains, *cis*-acting elements, and tissue-specific expression studies. The qRT-PCR analysis revealed that *PeWNK5*, *PeWNK7*, *PeWNK8,* and *PeWNK11* are involved in circadian rhythms. Transcriptome analysis of different abiotic stresses and co-expression analysis also revealed that *PeWNK8* and *PeWNK9* are involved in abiotic stress response. Thus, these genes can be used as good candidates for the production of genetically modified and economically important bamboo plants.

## Supplemental Information

10.7717/peerj.12718/supp-1 Supplemental Information 1 List of WNK genes used in this study and their conserved motifs

10.7717/peerj.12718/supp-2 Supplemental Information 2 List of cis-elements for WNK genes used in this study

10.7717/peerj.12718/supp-3 Supplemental Information 3 List of WNK genes contains the cis-elements (ABRE, GARE-motif, P-box, TATA-box, TCA-elements, Circadian, GC-motif and Sp1)

10.7717/peerj.12718/supp-4 Supplemental Information 4 Conserved domains of PeWNK genes

10.7717/peerj.12718/supp-5 Supplemental Information 5 List of genes co-expressed with PeWNK genes

10.7717/peerj.12718/supp-6 Supplemental Information 6 Details of qPCR primers

10.7717/peerj.12718/supp-7 Supplemental Information 7 The physical and chemical properties of WNK protein in Bambusoideae

10.7717/peerj.12718/supp-8 Supplemental Information 8 PeWNK genes location on the scaffolds

10.7717/peerj.12718/supp-9 Supplemental Information 9 The conserved GC-motif and Sp1 cis-elements distribution in the promoters of WNK genes

10.7717/peerj.12718/supp-10 Supplemental Information 10 Multiple sequence alignment between PeWNK protein sequences Conserved domains, motif and secondary structural arrangements were highlighted. The phosphorylation sites were mentioned in the yellow background.

## Additional Information and Declarations

### Data Availability

The following information was supplied regarding data availability: The raw data is available in the Supplementary File. The raw reads are available at GenBank: GSE169067.
