# Supplementary material for: Genome-wide identification and evolution of WNK kinases in Bambusoideae and transcriptional profiling during abiotic stress in Phyllostachys edulis
Source: PeerJ. 2022 Jan 13;10:e12718. doi: 10.7717/peerj.12718 (PMC8761366; doi:10.7717/peerj.12718)

**File S4: Conserved domains of PeWNK genes**

PeWNK2 protein conserved domains


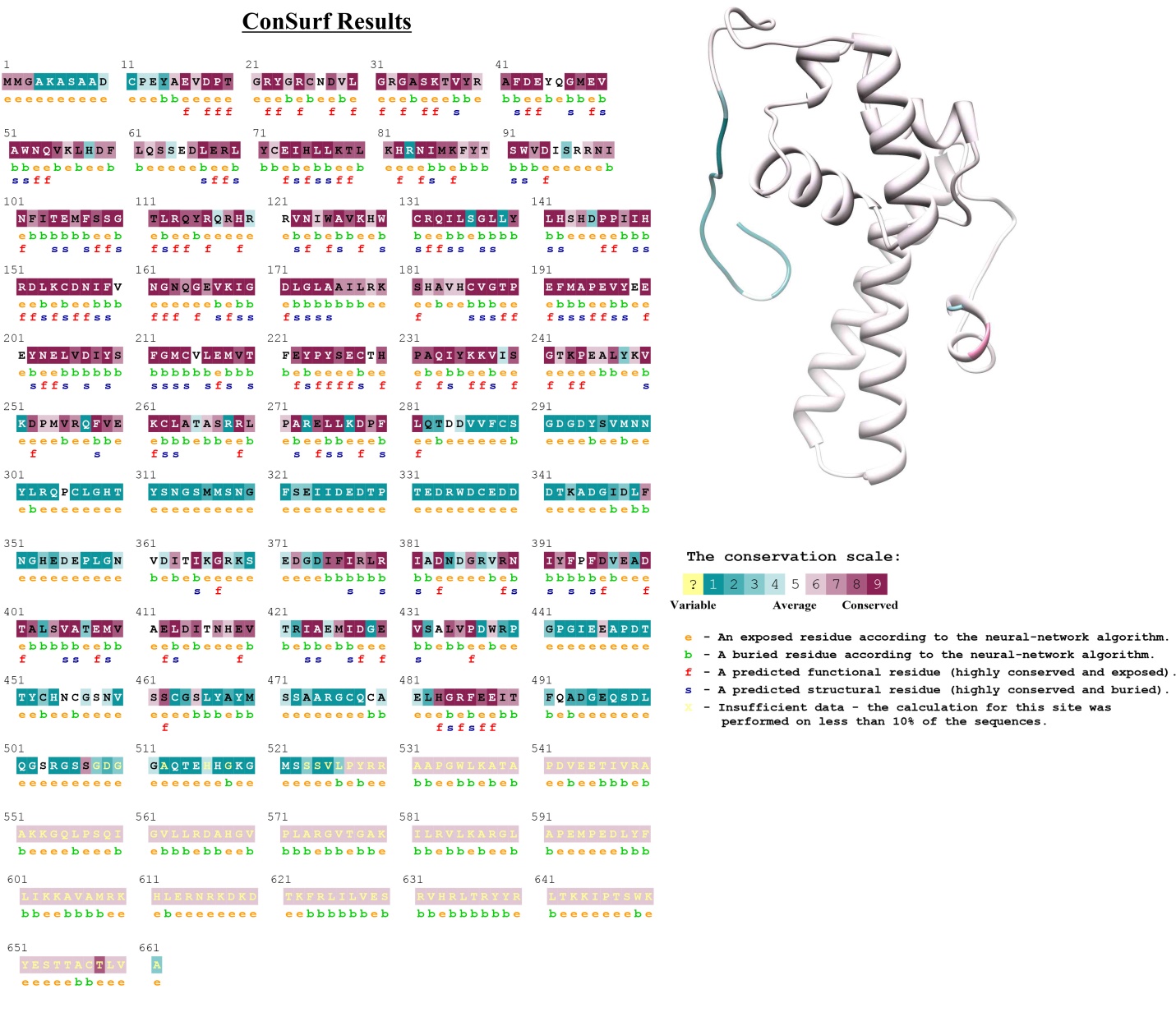


PeWNK3 protein conserved domains


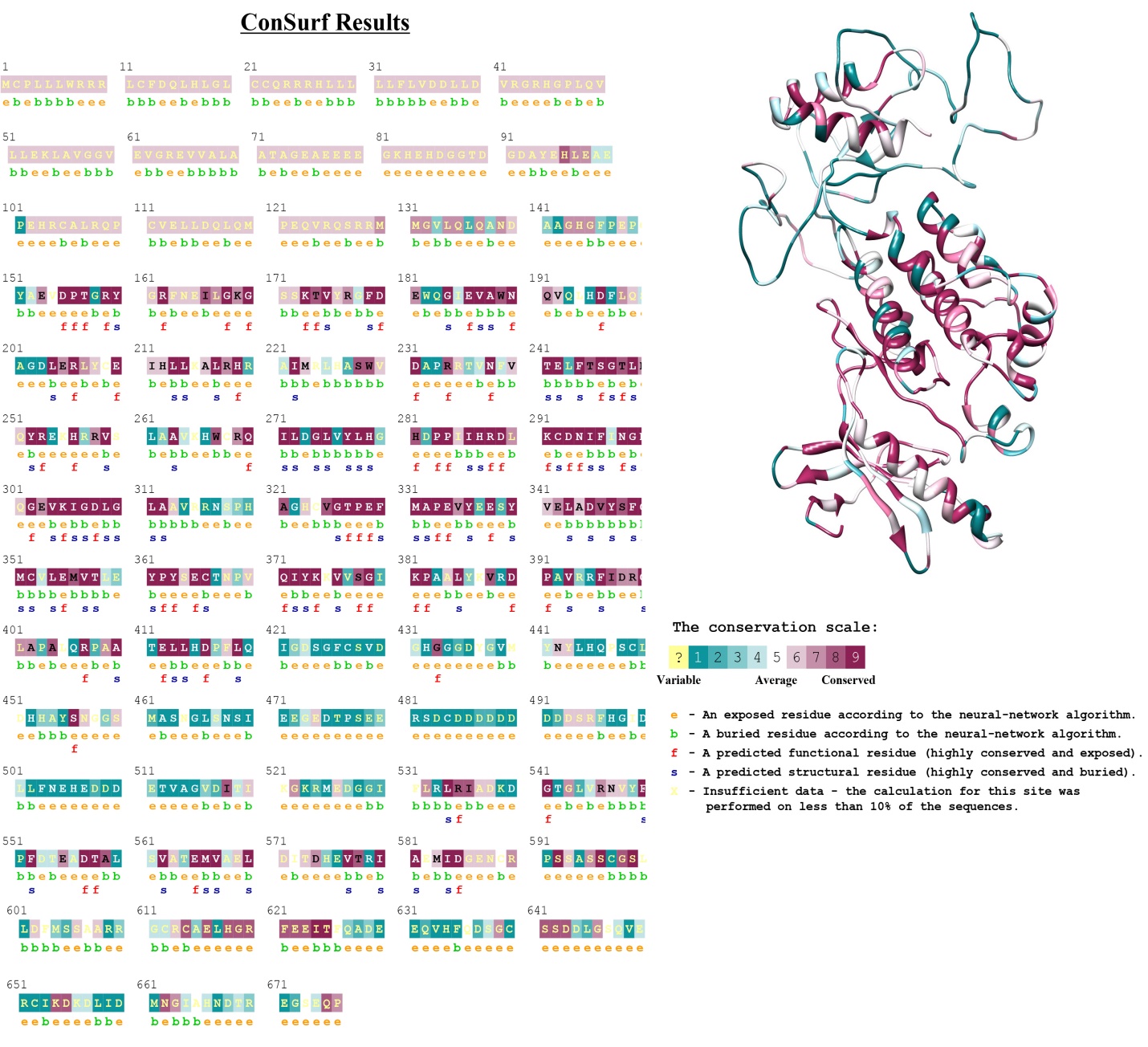


PeWNK4 protein conserved domains


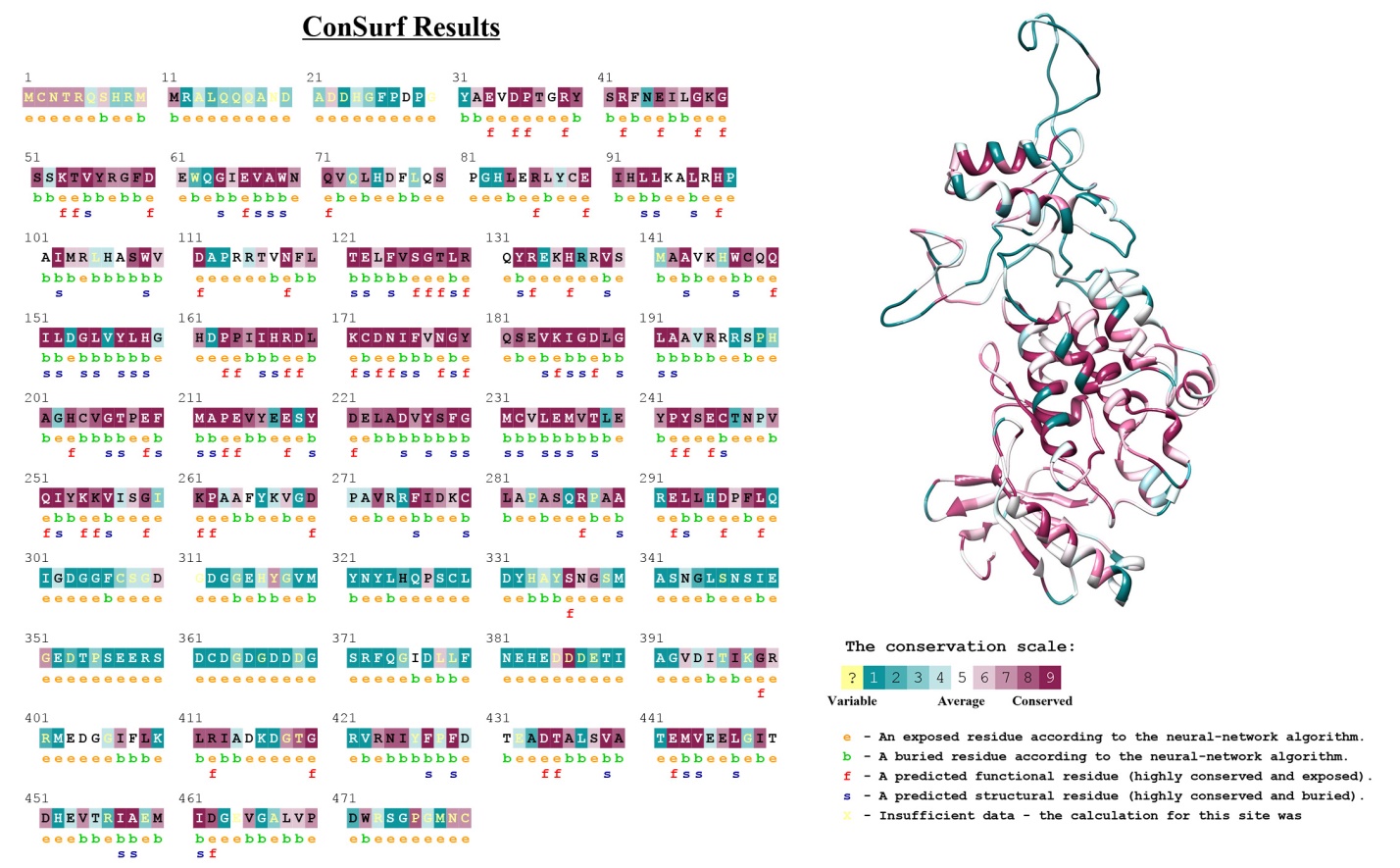


PeWNK5 protein conserved domains


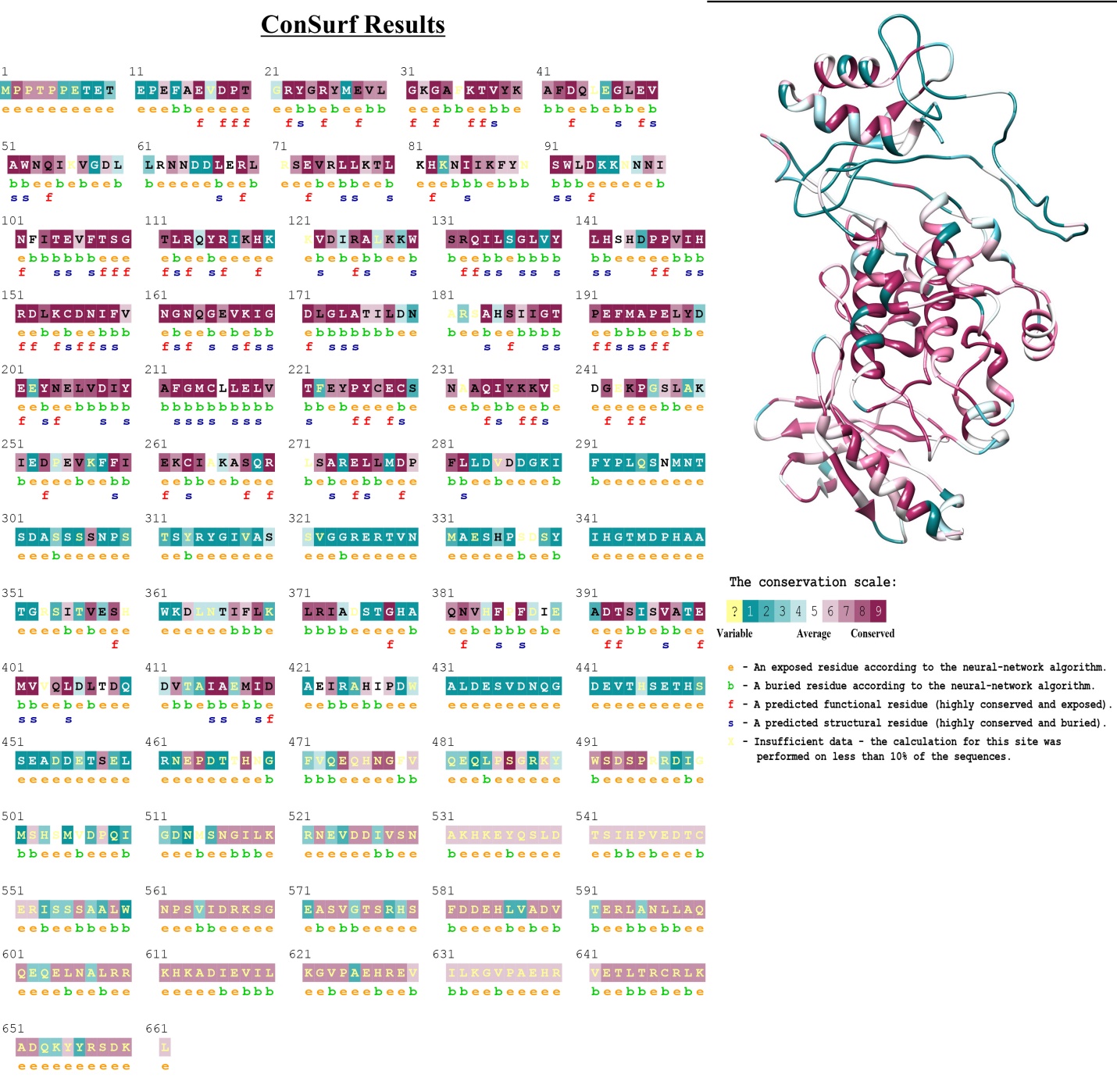


PeWNK6 protein conserved domains


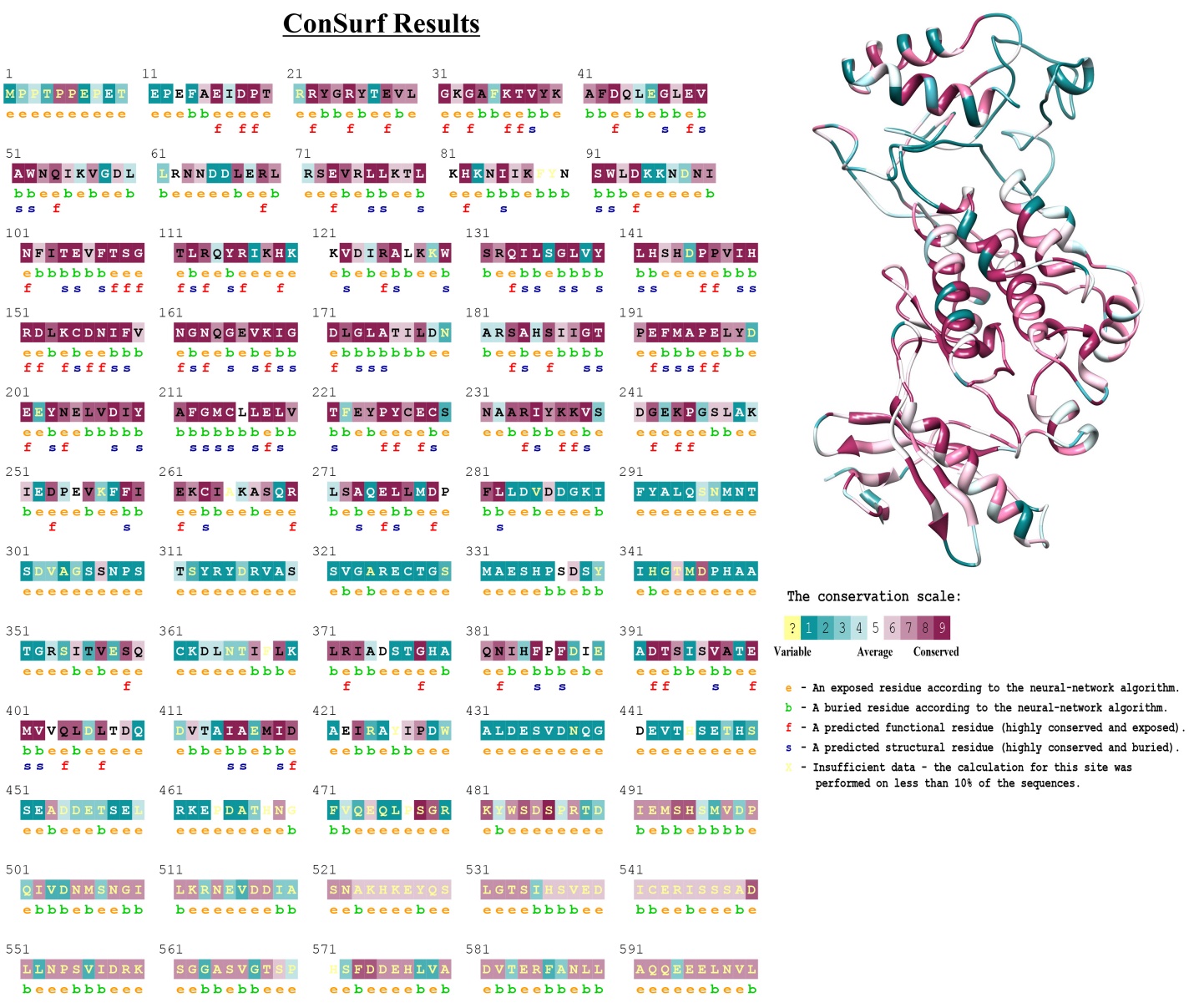


PeWNK7 protein conserved domains


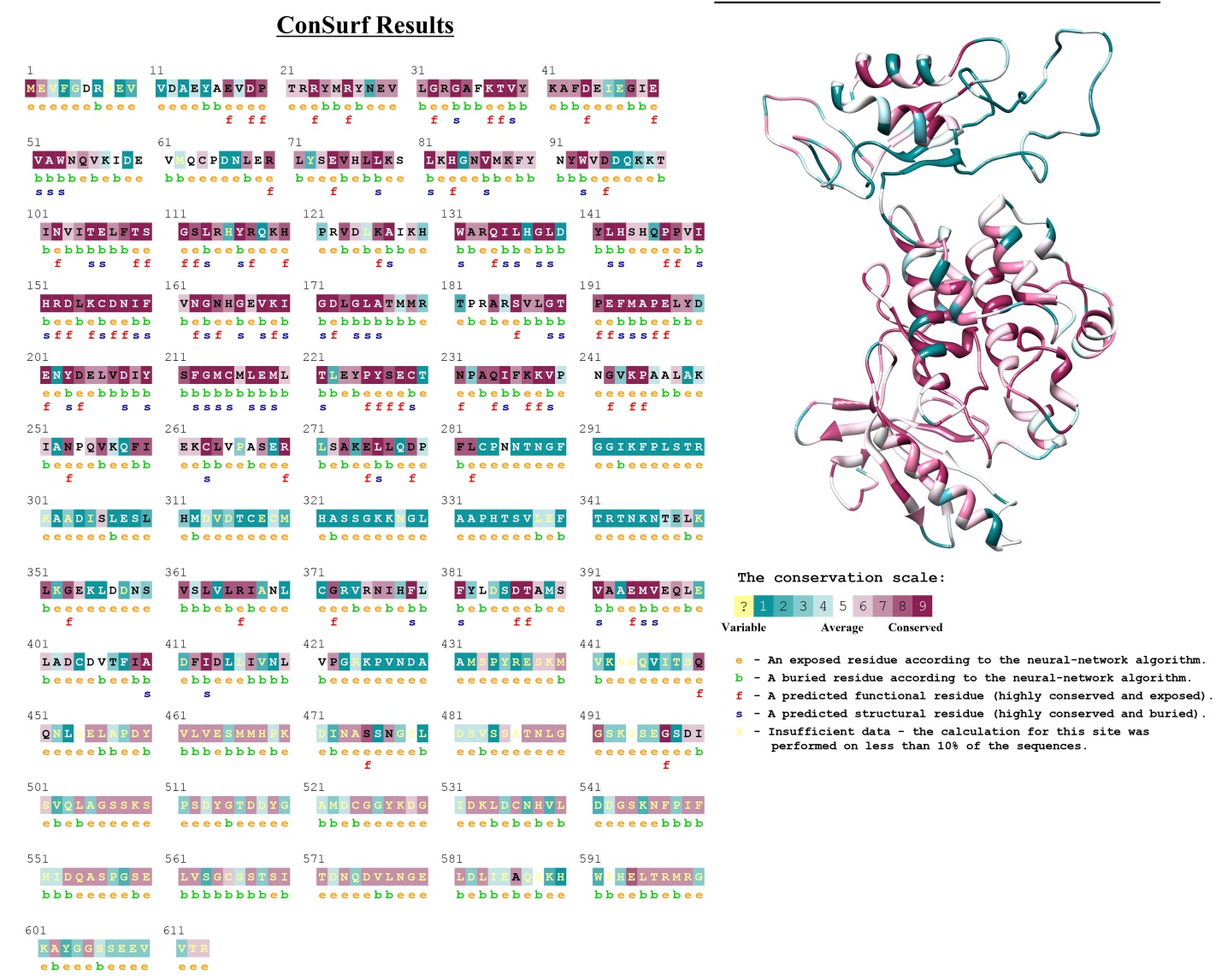


PeWNK8 protein conserved domains


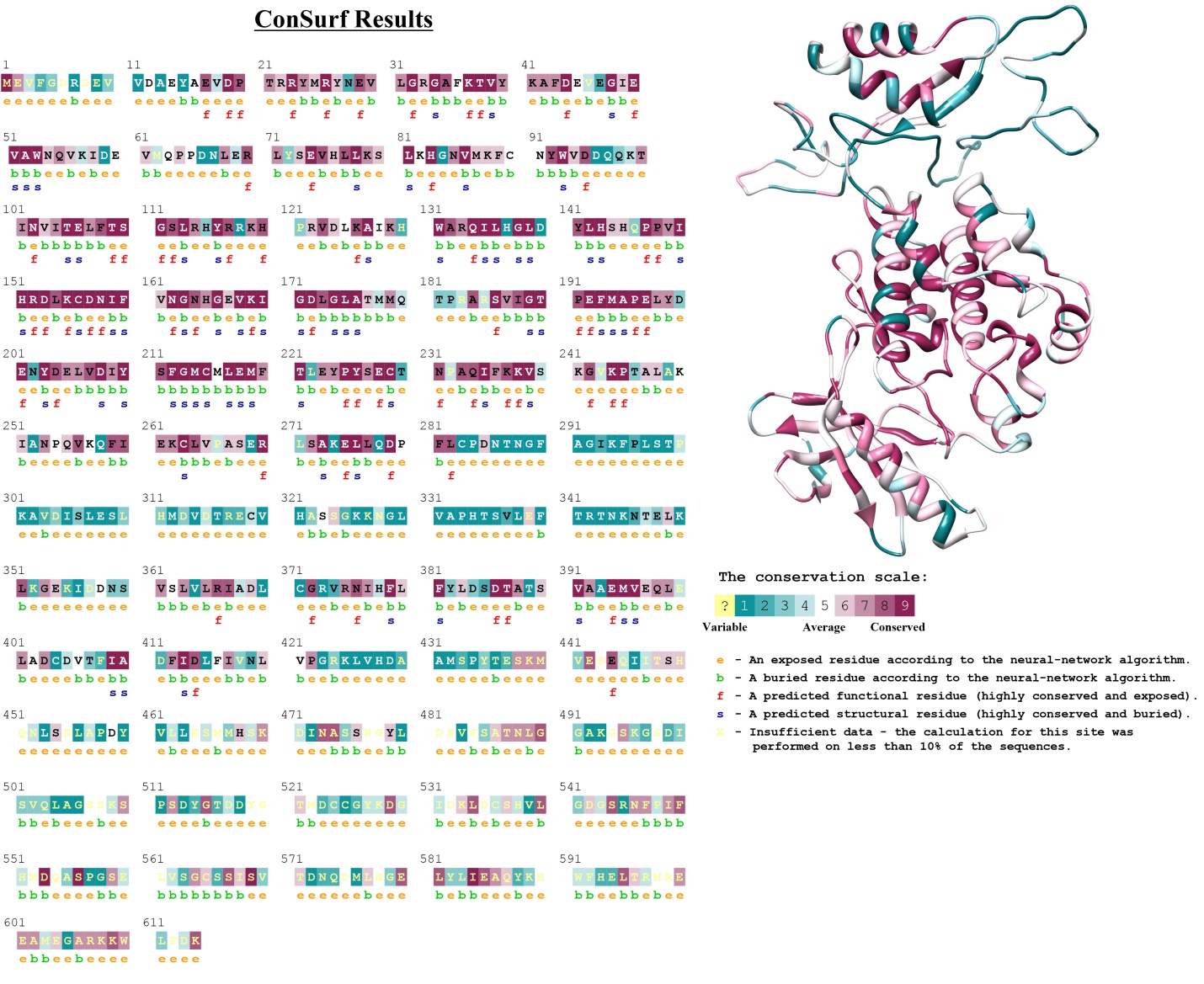


PeWNK9 protein conserved domains


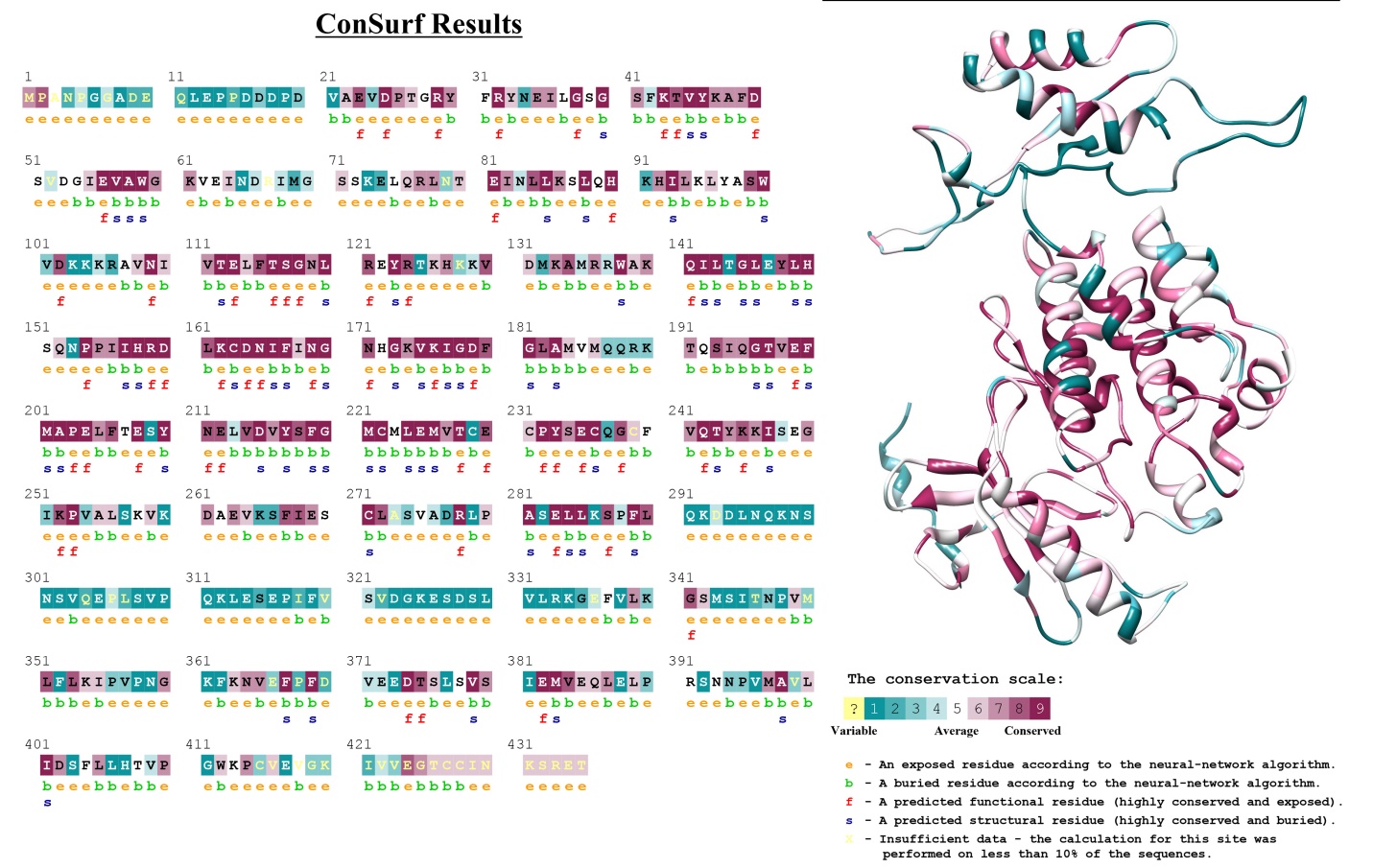


PeWNK10 protein conserved domains


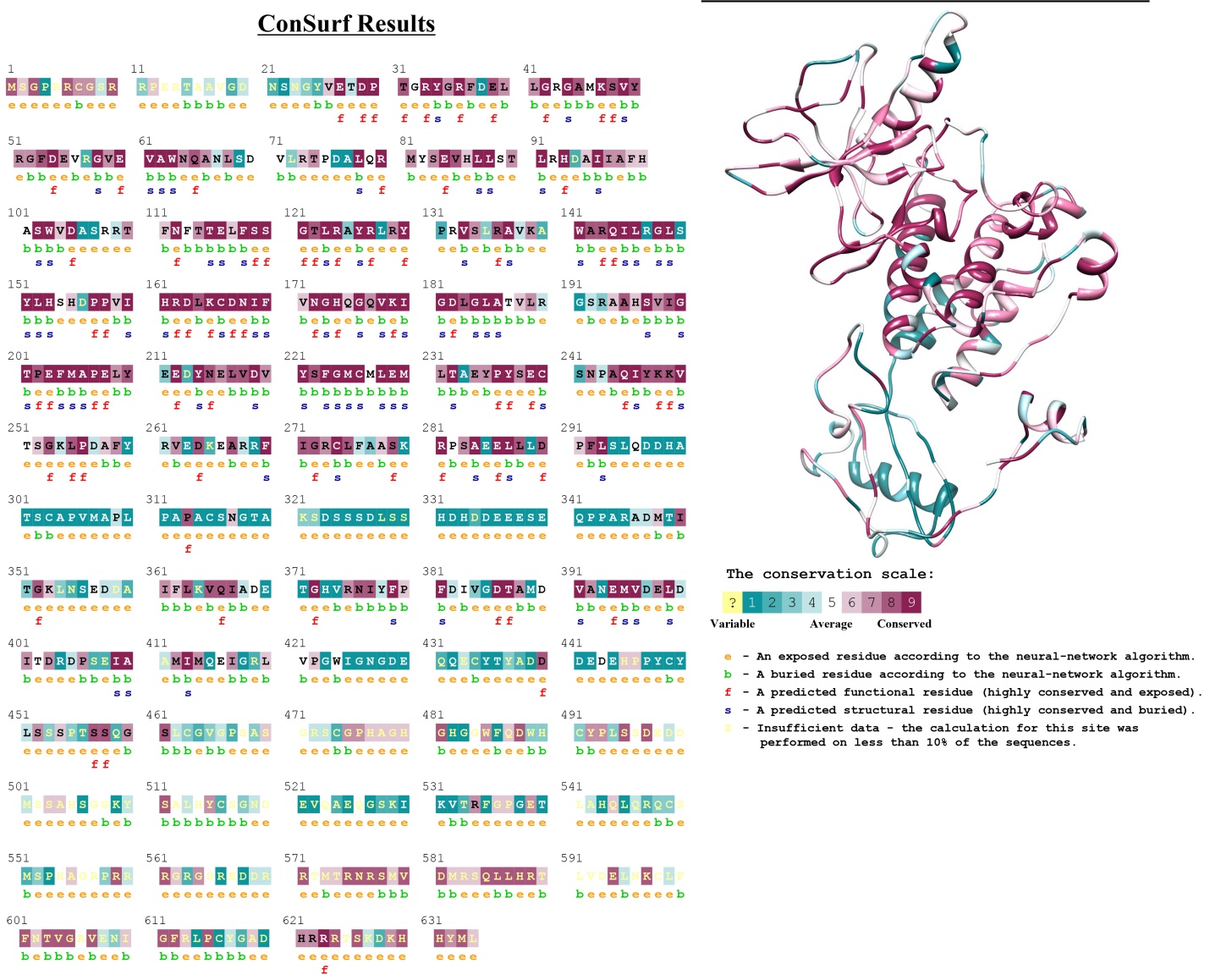


PeWNK11 protein conserved domains


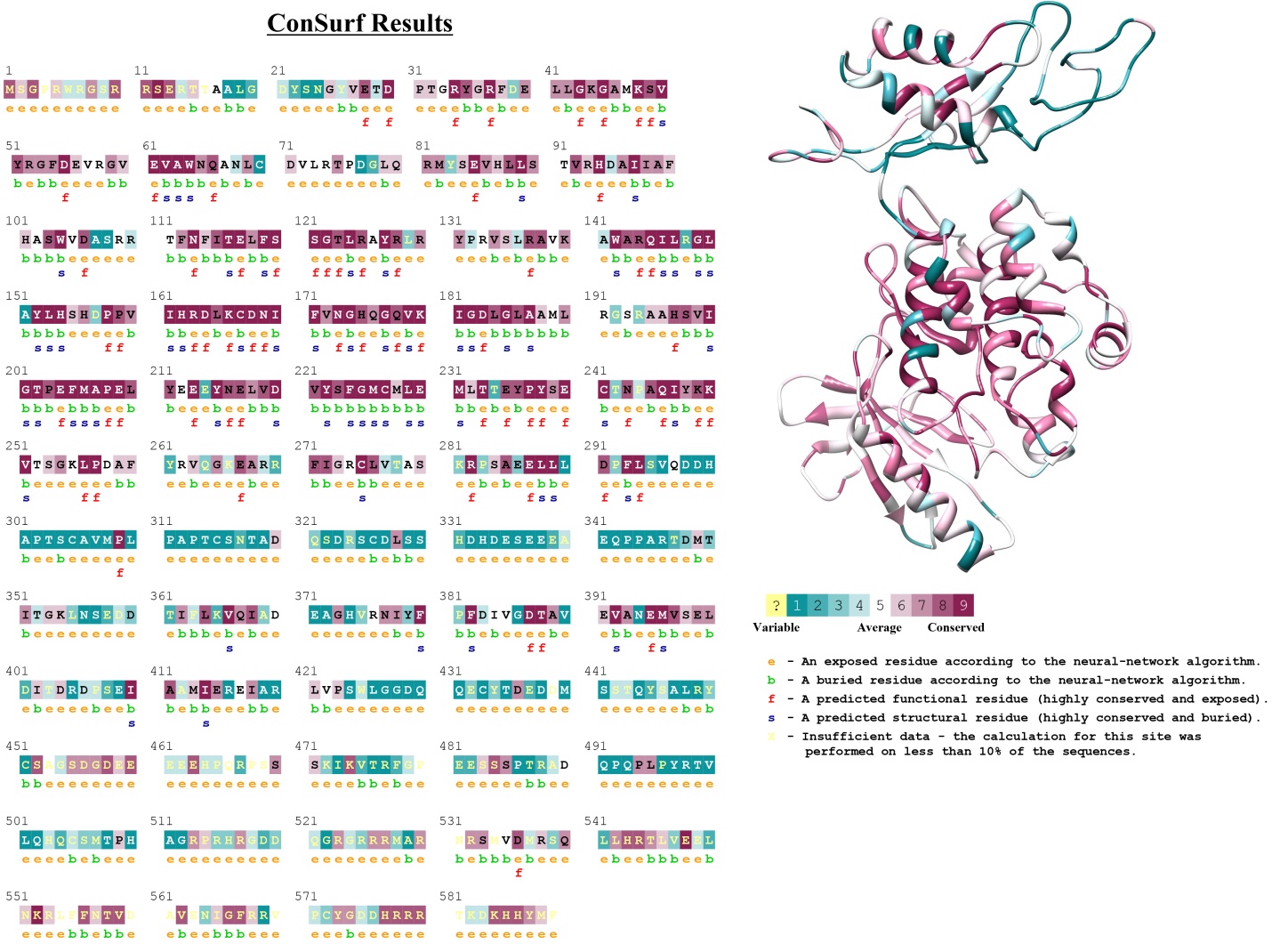

Supplement: Supplemental Information 4 [file peerj-10-12718-s004.docx]
