# Supplementary material for: Genome-wide identification and evolution of WNK kinases in Bambusoideae and transcriptional profiling during abiotic stress in Phyllostachys edulis
Source: PeerJ. 2022 Jan 13;10:e12718. doi: 10.7717/peerj.12718 (PMC8761366; doi:10.7717/peerj.12718)
Supplement: Supplemental Information 7 [file peerj-10-12718-s007.docx]

Table. S2. The physical and chemical properties of WNK protein in Bambusoideae

| S. NO | Gene ID | Number of amino acids | Molecular weight | Theoretical pI | instability index (II) | Aliphatic index | Grand average of hydropathicity (GRAVY) |
| --- | --- | --- | --- | --- | --- | --- | --- |
| 1 | BamWNK1 | 608 | 68170.61 | 5.12 | 34.32 | 88.32 | -0.261 |
| 2 | BamWNK2 | 567 | 63012.97 | 4.64 | 43.76 | 72.24 | -0.52 |
| 3 | BamWNK3 | 739 | 83597.78 | 5.78 | 37.39 | 91.58 | -0.211 |
| 4 | BamWNK4 | 290 | 32862.43 | 6.06 | 32.61 | 89.07 | -0.311 |
| 5 | BamWNK5 | 646 | 72779.41 | 5.18 | 42.5 | 81.66 | -0.561 |
| 6 | BamWNK6 | 718 | 79388.06 | 5.97 | 54.75 | 73.29 | -0.473 |
| 7 | BamWNK7 | 443 | 50124.4 | 5.28 | 43.95 | 87.16 | -0.223 |
| 8 | BamWNK8 | 701 | 79206.73 | 5.56 | 45.36 | 68.32 | -0.597 |
| 9 | BamWNK9 | 641 | 72209.89 | 5.08 | 45.09 | 81.83 | -0.555 |
| 10 | BamWNK10 | 290 | 32864.42 | 5.91 | 34.43 | 90.76 | -0.293 |
| 11 | BamWNK11 | 535 | 59549.88 | 4.76 | 41.27 | 60.56 | -0.636 |
| 12 | BamWNK12 | 653 | 72824.96 | 5.87 | 54.42 | 65.79 | -0.647 |
| 13 | BamWNK13 | 612 | 68126.59 | 5.77 | 40.28 | 84.26 | -0.304 |
| 14 | GanWNK1 | 560 | 62664.77 | 4.77 | 32.17 | 84.91 | -0.292 |
| 15 | GanWNK2 | 287 | 32350.8 | 5.81 | 31.43 | 88.29 | -0.298 |
| 16 | GanWNK3 | 619 | 69128.33 | 5.21 | 31.17 | 83.42 | -0.305 |
| 17 | GanWNK5 | 659 | 74133.85 | 5.34 | 46.81 | 64.55 | -0.62 |
| 18 | GanWNK6 | 285 | 32129.57 | 5.95 | 29 | 88.91 | -0.288 |
| 19 | OlaWNK1 | 602 | 67887.67 | 4.93 | 48.46 | 82.13 | -0.582 |
| 20 | OlaWNK2 | 593 | 66132.66 | 5.59 | 59.63 | 67.18 | -0.582 |
| 21 | OlaWNK3 | 702 | 79450.85 | 5.32 | 50.3 | 66.95 | -0.604 |
| 22 | OlaWNK4 | 598 | 67134.14 | 5.06 | 36.5 | 87.36 | -0.28 |
| 23 | OlaWNK5 | 317 | 35449.28 | 6.74 | 40.16 | 80.6 | -0.39 |
| 24 | OlaWNK6 | 581 | 63632.44 | 4.72 | 43 | 69.5 | -0.483 |
| 25 | RguWNK1 | 257 | 29047.42 | 6.73 | 32.74 | 95.95 | -0.173 |
| 26 | RguWNK2 | 507 | 56451.62 | 4.56 | 31.58 | 87.46 | -0.212 |
| 27 | RguWNK3 | 474 | 51616.74 | 5.22 | 43.02 | 69.96 | -0.395 |
| 28 | RguWNK4 | 686 | 77800.95 | 5.36 | 51.19 | 65.1 | -0.62 |
| 29 | RguWNK5 | 610 | 68917.94 | 5.07 | 50.97 | 86.77 | -0.376 |
| 30 | RguWNK6 | 575 | 63427.14 | 6.32 | 58.52 | 69.15 | -0.458 |
| 31 | PeWNK1 | 695 | 78301.6 | 5.42 | 47.2 | 68.06 | -0.585 |
| 32 | PeWNK2 | 661 | 74551.52 | 5.94 | 42.37 | 78.05 | -0.435 |
| 33 | PeWNK3 | 1842 | 150758.29 | 4.99 | 35.26 | 29.37 | 0.672 |
| 34 | PeWNK4 | 1845 | 151127.7 | 4.99 | 36.77 | 29.05 | 0.667 |
| 35 | PeWNK5 | 661 | 74646.51 | 5.21 | 41.59 | 81.42 | -0.565 |
| 36 | PeWNK6 | 640 | 72082.66 | 5.01 | 44.48 | 81.98 | -0.538 |
| 37 | PeWNK7 | 1770 | 145688.12 | 4.87 | 55.35 | 20 | 0.977 |
| 38 | PeWNK8 | 1905 | 157857.24 | 4.85 | 59.5 | 19.42 | 0.967 |
| 39 | PeWNK9 | 676 | 75639.72 | 4.92 | 45.25 | 81.79 | -0.425 |
| 40 | PeWNK10 | 480 | 53836.25 | 5.03 | 45.77 | 74.35 | -0.467 |
| 41 | PeWNK11 | 435 | 48915.35 | 5.7 | 42.08 | 87.06 | -0.283 |
