# Supplementary figures and images for: Genome-wide identification and evolution of WNK kinases in Bambusoideae and transcriptional profiling during abiotic stress in Phyllostachys edulis
Source: PeerJ. 2022 Jan 13;10:e12718. doi: 10.7717/peerj.12718 (PMC8761366; doi:10.7717/peerj.12718)

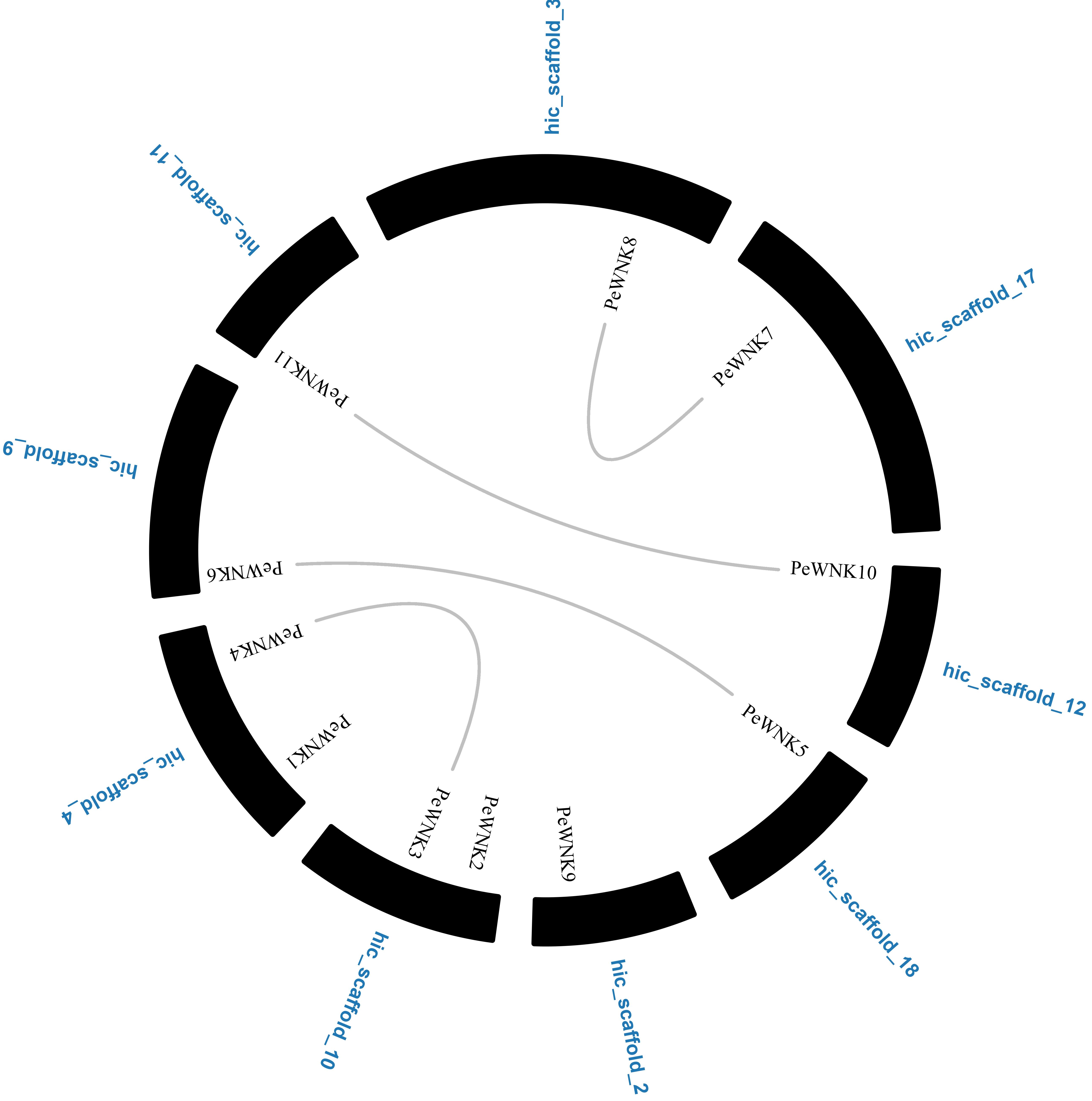

Supplement: Supplemental Information 8 [file peerj-10-12718-s008.jpg]

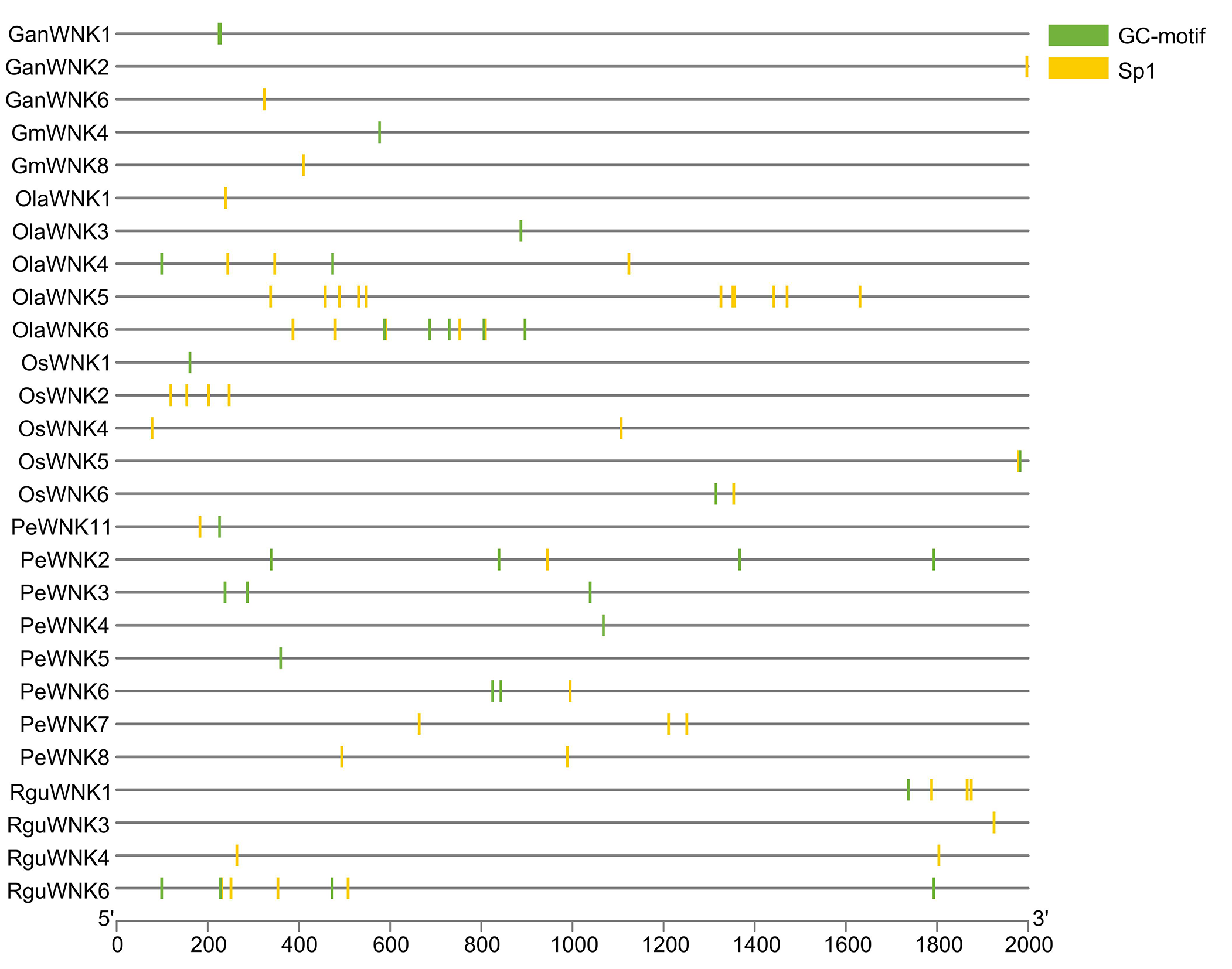

Supplement: Supplemental Information 9 [file peerj-10-12718-s009.png]

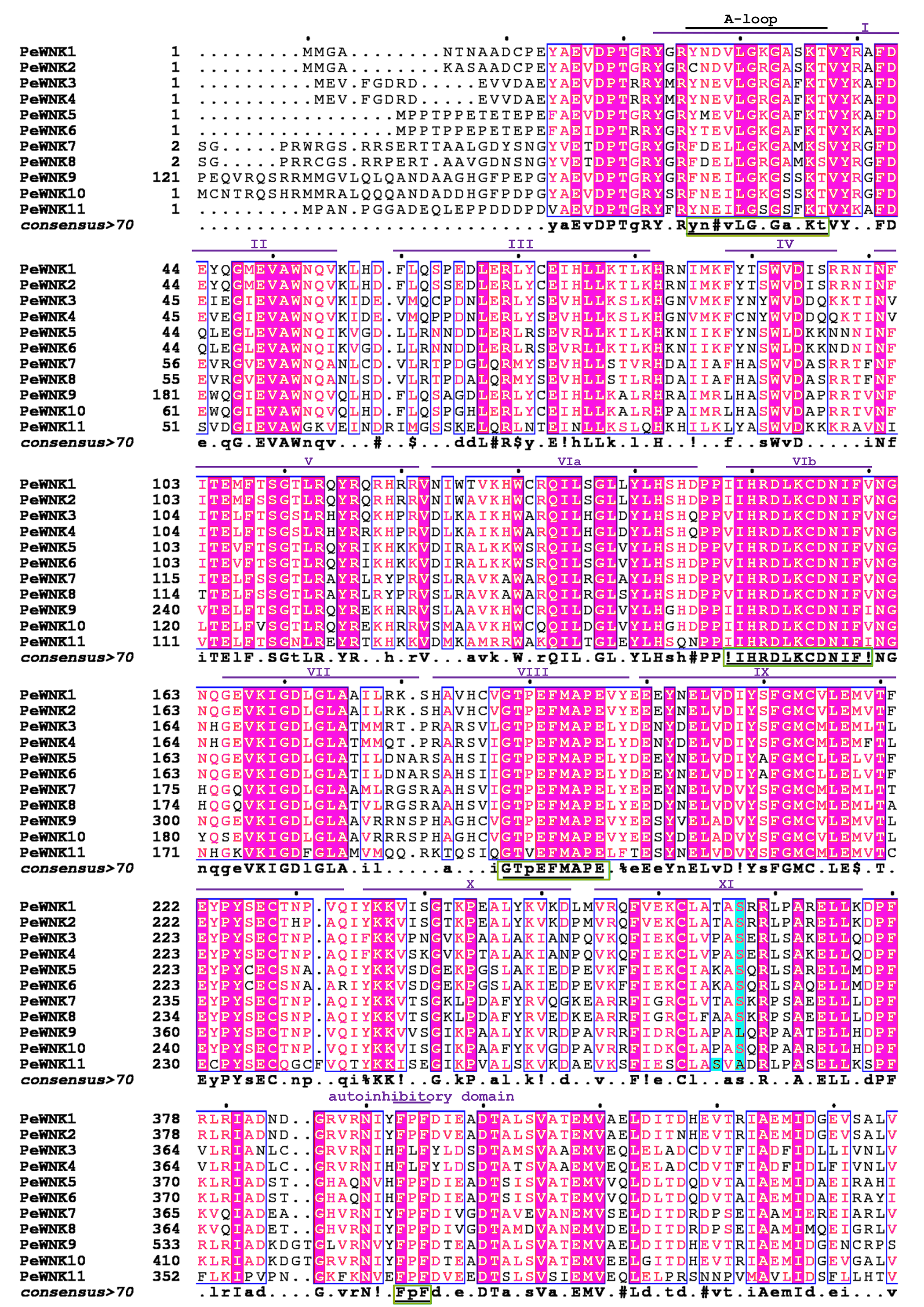

Supplement: Supplemental Information 10 — Conserved domains, motif and secondary structural arrangements were highlighted. The phosphorylation sites were mentioned in the yellow background. [file peerj-10-12718-s010.png]
